# Bone tissue engineering scaffolding: computer-aided scaffolding techniques

**DOI:** 10.1007/s40204-014-0026-7

**Published:** 2014-07-17

**Authors:** Boonlom Thavornyutikarn, Nattapon Chantarapanich, Kriskrai Sitthiseripratip, George A. Thouas, Qizhi Chen

**Affiliations:** 1 Department of Materials Engineering, Monash University, Clayton, VIC 3800 Australia; 2 Department of Mechanical Engineering, Faculty of Engineering at Si Racha, Kasetsart University, 199 Sukhumvit Road, Si Racha, Chonburi 20230 Thailand; 3 National Metal and Materials Technology Center (MTEC), 114 Thailand Science Park, Phahonyothin Road, Klong Luang, Pathumthani 12120 Thailand

**Keywords:** Computer-aided scaffolding techniques, Solid free-form fabrication, Bioceramics, Bone tissue engineering, Scaffold

## Abstract

Tissue engineering is essentially a technique for imitating nature. Natural tissues consist of three components: cells, signalling systems (e.g. growth factors) and extracellular matrix (ECM). The ECM forms a scaffold for its cells. Hence, the engineered tissue construct is an artificial scaffold populated with living cells and signalling molecules. A huge effort has been invested in bone tissue engineering, in which a highly porous scaffold plays a critical role in guiding bone and vascular tissue growth and regeneration in three dimensions. In the last two decades, numerous scaffolding techniques have been developed to fabricate highly interconnective, porous scaffolds for bone tissue engineering applications. This review provides an update on the progress of foaming technology of biomaterials, with a special attention being focused on computer-aided manufacturing (Andrade et al. 2002) techniques. This article starts with a brief introduction of tissue engineering (Bone tissue engineering and scaffolds) and scaffolding materials (Biomaterials used in bone tissue engineering). After a brief reviews on conventional scaffolding techniques (Conventional scaffolding techniques), a number of CAM techniques are reviewed in great detail. For each technique, the structure and mechanical integrity of fabricated scaffolds are discussed in detail. Finally, the advantaged and disadvantage of these techniques are compared (Comparison of scaffolding techniques) and summarised (Summary).

## Bone tissue engineering and scaffolds

Tissue engineering is defined as a multidisciplinary scientific branch that combines cell biology, materials science and engineering, and regenerative medicine (Langer and Vacanti 1993). This innovative technology has attracted increasing attention as an alternative strategy to treat damaged organs and tissues that cannot be self-regenerated, such as full-thickness skin burn, over critical-sized bone defects, and chronic cartilage disease. Tissue engineering aims to eliminate the disadvantages of the conventional clinical treatments (Burg et al. 2000) associated with donor-site morbidity and scarcity in autografting and allografting (allografting also introduces the risk of disease and infection transmission). Developed as an artificial bone matrix, a tissue engineering scaffold plays an essential role in regenerating bone tissue.

In general, a tissue engineering process begins with the fabrication of a biologically compatible scaffold that will support living cells for their attachment, proliferation and differentiation, and thus promote tissue regeneration both in vitro and in vivo. Ideally, a tissue engineering scaffold should be biocompatible, biodegradable, highly porous and interconnected, and mechanically reliable. To engineer bone, which is a vascularised tissue, a well-interconnected porosity is highly desirable for the sake of vascularisation. Appropriate mechanical strength is another important requirement for implants at load-bearing sites. The specific criteria of an ideal scaffold in bone tissue engineering are summarised in Table 1.

**Table 1:** Criteria of an ideal scaffold for bone tissue engineering (Bruder and Caplan 2000; Chen et al. 2008; Liu et al. 2013)

Criteria	Requirement
Biocompatibility	Support and foster cells’ attachment, proliferation and differentiation, and initiate tissue regeneration both in vitro and in vivo
Osteoconductivity	Encourage host bone adherence and growth into the scaffold
Biodegradability	Be able to degrade at a physiologically relevant rate
Mechanical properties	Maintain proper mechanical stability for tissue regeneration
Porous structure	Be highly porous (>90 %) and interconnected, with pore diameters between 300 and 500 μm, to allow cells to penetrate into a pore structure, and promote new bone formation, as well as vascularisation. It must be able to deliver nutrients into the scaffold and transport undesirable metabolites outside scaffold
Fabrication	Possess desired fabrication capabilities (e.g. being readily produced into irregular shapes of scaffolds that match the defects in the bone of individual patients)
Commercialisation	Be fabricated at an acceptable cost for commercialisation

## Biomaterials used in bone tissue engineering

The selection and design of a bone matrix-like biomaterial are primarily determined by the composition of the osseous tissue. The extracellular matrix (ECM) of bone is a composite that primarily comprises hydroxyapatite (HA) (biological ceramics) embedded within a collagen matrix (biological polymers) and water. Table 2 provides the composition of the natural bone matrix. Not surprisingly, scaffolding biomaterials applied to bone tissue engineering are principally made from (1) natural or synthetic polymers, (2) ceramics or (3) their composites aimed at mimicking the composition and structure of natural bone (Vacanti 2000; Correlo et al. 2011; Wolfe et al. 2011; Reichert and Hutmacher 2011). For this reason, this section is devoted to a concise review on these promising scaffolding biomaterials, focusing on biocompatibility, biodegradability, and mechanical properties, which are the most important factors to consider in the development of a bone substitute.

**Table 2:** Composition of natural bone matrix

Composition	Content and function
Biological ceramic	Carbonated HA Ca_10_(PO_4_)_6_(OH)_2_ accounts for approximately 70 % of the weight of bone. The inorganic component provides compressive stiffness to bone
Biological polymer	Roughly one-third of the weight of bone is composed of the organic matter, which is primarily type I collagen and ground substance. Type I collagen fibres are elastic and flexible, and thus tolerate stretching, twisting, and bending. Bone collagen differs slightly from soft-tissue collagen of the same type in *having a great number of intermolecular cross*-*links*. Ground substance contains proteoglycans aggregates and several specific structural glycoproteins

### Polymeric materials

#### Naturally derived biopolymers

Much research effort has been invested in the fabrication of scaffolds from naturally derived biopolymers, including collagen, demineralised ECM-based materials, and chitosan and its derivative for the purpose of bone tissue engineering. Due to their excellent biocompatibility, naturally derived biopolymers generally do not cause significant inflammatory responses when implanted into the body.

Collagen and ECM-degenerated proteins (i.e. gelatine) have gained early attention as biomaterials used for bone tissue engineering due to their advantages, such as excellent biocompatibility, biodegradability and cell-binding properties (Burg et al. 2000; Russell and Block 1999; Dawson et al. 2008; Eslaminejad et al. 2007; Sharifi et al. 2011). However, there are serious concerns associated with the immunogenicity, rapid degradation, and poor mechanical properties of collagen. To minimise these drawbacks, efforts have been invested in the development of chemical cross-linked collagen combined with synthetic polymers (Ferreira et al. 2012; Wojtowicz et al. 2010). Chitosan and its derivative are another group of natural biopolymers. They have been widely explored for bone tissue engineering because of their hydrophilic surfaces that promote cell attachment, proliferation and differentiation (Brown and Hoffman 2002; Thein-Han and Misra 2009). In addition to the enhanced osteoconductivity (the process in which growth of bone on the biomaterial surface) in vivo, chitosan also exhibits an ability to entrap growth factors at the wound site (Muzzarelli et al. 1993; Muzzarelli and Muzzarelli 2005).

#### Synthetic polymers

Although the naturally derived biopolymers offer benefits as mentioned above, their use may be limited owing to poor mechanical properties and a high degradation rate. Following efforts using naturally occurring polymers as scaffolds, attention has been paid to synthetic polymers. Besides being biocompatible and biodegradable, synthetic polymers offer advantages over the biologically derived biopolymers. These include controllable degradation rate, predictable and reproducible mechanical properties, and ease of fabrication with tailorable shapes and sizes as required (Wolfe et al. 2011; Vacanti et al. 2000; Middleton and Tipton 2000; Puppi et al. 2010; Dhandayuthapani et al. 2011). Further, synthetic polymers have a long shelf life and can be sterilised. However, they may involve shortcomings such as eliciting persistent inflammatory reactions when eroded, or they may be mechanically incompliant or unable to integrate with host tissues. It has been envisaged that such shortcomings might be overcome by selecting an appropriate synthetic biopolymer and by the modification and functionalization of their structures for the specific tissue engineering purposes (Tian et al. 2012).

The degradable synthetic polymers, which have widely been used as scaffolding materials in bone tissue engineering, are polyesters. Polyesters are characterised by the ester functional groups along their backbones, which are formed via the condensation polymerisation between carboxylic acid group (–COOH) and a hydroxyl group (–OH) on the precursor monomers. Two widely used monomers are lactic acid and glycolic acid. These small precursor molecules are endogenous to the human metabolism. In principle, polyesters can degrade to natural metabolic products through hydrolysis. Saturated poly(α-hydroxy esters) such as poly(lactic acid) (PLA), poly(glycolic acid) (PGA), poly($\varepsilon$-caprolactone) (PCL), and their copolymers have been extensively investigated (Mano et al. 2004; Kohn 1996; Rezwan et al. 2006).

PLA was the first polyester studied for application in tissue engineering because of its biocompatibility and biodegradability. It has three stereoisomers: poly(_L_-lactic acid) (PLLA), poly(_D_-lactic acid) (PDLA), and poly(_D,L_-lactic acid) (PDLLA). Among these stereoisomers, PDLLA is of particular interest for scaffold production in bone tissue engineering application, because it possesses excellent biocompatibility in vivo and good osteoinductivity (the process of stimulating the proliferation and differentiation of progenitor or osteogenic cells) (Schmidmaier et al. 2001).

PGA is employed as a scaffolding material because of its relatively hydrophilic nature. Both PLA and PGA undergo bulk erosion via ester linkage hydrolysis into the degradation products, lactic acid or glycolic acid that are natural metabolites. However, PGA degrades rapidly in aqueous solution and the in vivo environment, being completely resorbed within 4–6 months, which leads to premature mechanical failures of scaffolds (Wolfe et al. 2011; Ma and Langer 1995; Langer et al. 1995). Hence, PGA alone is limited for use in scaffolds for bone tissue engineering. The degradation rates of PLA and PGA can be ranked in the following order (Rezwan et al. 2006). 

PCL is similar to PLA and PGA but it has a much slower degradation rate, primarily due to its high crystallinity. Owing to the ability to promote osteoblast growth and maintain its phenotype, PCL scaffold has been used as a long-term implant in the field of bone tissue engineering (Woodruff and Hutmacher 2010; Pitt et al. 1981; Rich et al. 2002). However, the synthesis of PCL with other fast-degradable polymers can tune degradation kinetics of these polymers. Selected physical properties of the polyesters being discussed are listed in Table 3.

**Table 3:** Mechanical properties and degradation time of synthetic aliphatic polyesters (Rezwan et al. 2006)

Polymers	Tensile or compressive^a^ strength (MPa)	Modulus (Potijanyakul et al. 2010)	Degradation time (months)
PDLLA	Pellet: 35–150^a^	Film or disk: 1.9–2.4	12–16
	Film or disk: 29–35		
PLLA	Pellet: 40–120^a^	Film or disk: 1.2–3.0	>24
	Film or disk: 28–50	Fibre: 10–16	
	Fibre: 870–2,300		
PGA	Fibre: 340–920	Fibre: 7–14	6–12
PLGA	41.4–55.2	1.4–2.8	Adjustable
PCL	10–15	0.15–0.33	Bulk >24
P3HB	25–45	1.5–1.8	Very slow

These polyesters remain popular for a variety of reasons, predominantly excellent biocompatibility and biodegradability. These materials have chemical properties that allow hydrolytic degradation through de-esterification. Once degraded, the acidic products of each polymer can be metabolised through various physiological pathways by tissues. For example, PLA can be cleared through tricarboxylic acid cycle. Due to their degradation properties, these polymers have been used in medical devices approved by the United States Food and Drug Administration (FDA) for human clinical uses, such as surgical sutures. However, release of acidic degradation products can cause a severe inflammatory response in the body (Bergsma et al. 1993; Tam et al. 1996; Martin et al. 1996; Suuronen et al. 1998; Tatakis and Trombelli 1999; Bostman and Pihlajamaki 2000).

Since the 1990s, other types of aliphatic polyester: polyhydroxyalkanoates (PHA) particularly poly-3-hydroxybutyrate (P3HB), copolymer of 3-hydroxybutyrate and 3-hydroxyvalerate (PHBV), poly-4-hydroxybutyrate (P4HB), copolymers of 3-hydroxybutyrate and 3-hydroxyhexanoate (PHBHHx) and poly-3-hydroxyoctanoate (Leong et al. 2007) have been increasingly investigated as scaffolding materials for tissue engineering application due to their high biocompatibility (Chen and Wu 2005; Misra et al. 2006). They are natural thermoplastic polyesters produced by a wide variety of microorganisms under imbalanced growth conditions (Doi et al. 1995; Li et al. 2005). Their wide biodegradation kinetics can be tuned via thermal processing, and this makes PHAs attractive as biomaterials for a wider range of applications in medical devices.

The mechanical properties of PHAs can be widely adjusted by blending with either other polymers or inorganic materials to meet the specific requirements of different applications (Chen and Wu 2005; Doi et al. 1995). P3HB is a tough, brittle polymer, and an important member of the PHA family. This polymer degrades with no evidence of an undesirable chronic inflammatory response after up until 12 months after implantation (Doyle et al. 1991).

However, the limitation of some PHA polymers is their ineffective large-scale production and the time-consuming purification process from bacterial cultures that require an appropriate extraction system (Chen and Wu 2005; Verma et al. 2002). Hence, the challenge in their utility is to reduce the cost of production in the extraction procedure at an industrial scale. In general, the members of the PHA family degrade more slowly than PLA; typically, they take longer than 3 years. This low-degradation rate hampers their application in bone repair, which typically has a healing rate of several months.

#### Synthetic elastomers

Over the past 10 years, a number of research articles have reported on the development and clinical application of synthetic, biodegradable elastomeric biomaterials for tissue engineering applications (Chen et al. 2008, 2013). Elastomeric polymers (elastomers) have received increasing attention because they can provide mechanical stability and sustainable elasticity to tissues and organs without mechanical irritation to the host (Wang et al. 2002). Among the many elastomeric polymers, poly(glycerol sebacate) (PGS) is a tough, synthetic biodegradable cross-linked elastomer that has been extensively studied for use as a scaffolding biomaterial in tissue engineering applications and regenerative medicine (Bettinger 2011). It is synthesised through the polycondensation (esterification) reaction of tri-functional glycerol, HOCH_2_CH(OH)CH_2_OH, and di-functional sebacic acid (HOOC)(CH_2_)_8_(COOH), producing the pre-polymer that can be melt processed or organic solvent processed into various shapes. Then, this pre-polymer is reacted to form a three-dimensional (3D), loosely cross-linked polymer. Young’s modulus of PGS is in the range of 0.056–1.2 MPa, and its elongation at break ranges from 41 to 448 %, depending on the synthesis conditions, reported by Chen et al. (2008).

Chen’s investigation also reported that PGS had a wide range of degradation kinetics, which can be fine-tuned through polycondensation processing to match clinical requirements. Moreover, it showed good biocompatibility with several cell types. Another study by Li et al. (2013), investigating the influence of synthesis conditions on the mechanical properties and cytocompatibility of PGS, showed that the modulus and ultimate tensile strength increased with curing duration. In addition, the cell viability of mouse fibroblasts was better for PGS samples with a higher conversion. The in vivo evaluation showed that PGS has a favourable tissue response with significantly less inflammation in comparison with poly(α-hydroxy acid) (PLGA) (Sundback et al. 2005). Additionally, many investigations have demonstrated that this elastomer has an excellent biocompatibility in vivo for tissue engineering applications (Kemppainen and Hollister 2010; Stuckey et al. 2010).

However, the rapid degradation of PGSs is believed to limit their application for use as scaffolding materials in engineering tissues that typically have healing rates of several months or years. To overcome these limitations, making a composite with bioceramics of PGS could be a potential strategy. For example, the investigation of PGS-Bioglass^®^ composites developed by Liang et al. (2010) showed that the addition of Bioglass^®^ filler to PGS could be a control of degradation kinetics, which is independent of the mechanical properties of the composites. In addition, the composites have significantly improved biocompatibility compared with pure PGS.

### Bioceramics

Bioceramics can broadly be divided into calcium phosphates and bioactive glasses. This section provides a brief overview on bioceramics, and detailed reviews on most recent development of bioceramics can be found elsewhere (Chen et al. 2012).

#### Calcium phosphates

HA (Ca_10_(PO_4_)_6_(OH)_2_) and related calcium phosphate (Bruder and Caplan 2000)-based ceramics (e.g. β-tricalcium phosphate [$\beta$-TCP]) have been researched for biomedical applications (Hench and Wilson 1999; Chai et al. 2012). They have excellent biocompatibility due to their chemical and structural similarity to the mineral phase of human bones. These bioceramics are characterised by their bioactivity, an ability to bond directly to the surrounding bone tissue, and osteoconductivity, an ability to support osteoblastic cell attachment, proliferation and differentiation both in vivo and in vitro studies (Boccaccini and Blaker 2005). The principal disadvantage of the use of HA and related calcium phosphates as bone scaffold is that the slow degradation of these inorganic ceramics in the body limits their utility for bone-regeneration applications. Clinical investigation has shown that implanted HA and calcium phosphates are virtually inert, remaining within the body for as long as 6–7 years post-implantation (Marcacci et al. 2007). Clinical follow-up studies have demonstrated that there are no visible signs of biomaterial resorption (Marcacci et al. 2007). The dissolution rate of the HA and related calcium phosphates can be ranked in the following order (Rezwan et al. 2006):

Amorphous CaP>amorphous HA>crystalline CaP>crystalline HA.

HA and related calcium phosphates also have unsatisfactory mechanical properties. Compared with those of human bone, the compressive strength values of HA and related calcium phosphates are much higher; however, they fail in tensile strength and fracture toughness (Table 4). Therefore, the use of calcium phosphates alone is limited to non-load-bearing sites despite their good biocompatibility and osteoconductivity.

**Table 4:** Mechanical properties of calcium phosphate systems and human bone (Chen et al. 2012)

Ceramics	Compressive strength (MPa)	Tensile strength (MPa)	Elastic modulus (Potijanyakul et al. 2010)	Fracture toughness (MPa$\sqrt m$)
Calcium phosphates	20–900	30–200	30–103	<1.0
HA	>400	~40	~100	~1.0
45S5 Bioglass^®^	~500	42	35	0.5–1
Cortical bone	130–180	50–151	12–18	6–8

#### Bioactive glasses

The advantage of bioactive glasses over HA and related CaP is their degradability (Chen et al. 2012; Hench 2006; O’Donnell 2012; Jones 2013; Baino and Vitale-Brovarone 2011; Fu et al. 2011; Gerhardt and Boccaccini 2010). Many compositions of bioactive glasses have been developed; these can be grouped according to their chemistry: bioactive silicate (SiO_2_) glasses, bioactive phosphate (P_2_O_5_) glasses, and bioactive borate (B_2_O_3_) glasses (Jones 2013; Baino and Vitale-Brovarone 2011). This section focuses on the first category.

**Fig. 1:**
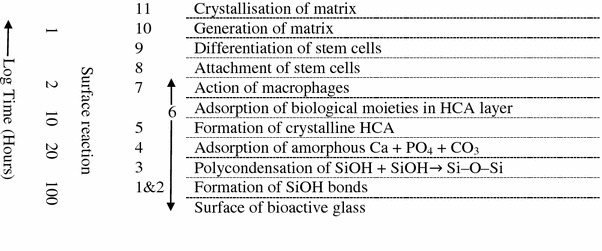
Sequence of interfacial reactions involved in forming a bond between bone and bioactive ceramics and glasses (O’Donnell 2012; Jones 2013; Gerhardt and Boccaccini 2010)

Bioactive silicate glass, such as 45S5 Bioglass^®^, was invented by Hench in 1969 (Hench 2006). The main components of bioactive silicate glasses are SiO_2_–Na_2_O–CaO–P_2_O_5_, having <55 % SiO_2_ in weight percentage. Bioactive silicate glasses are recognised as Class A bioactive materials because they offer high bioactivity involving both osteoconduction and osteoproduction, while HA is recognised as Class B bioactive material because it exhibits only osteoconductivity (Chen et al. 2008). Bioactive silicate glasses are able to induce a strong bond to bone tissue when implanted or exposed to physiological body fluid. The formation of a carbonated hydroxyapatite (HCA) layer on the surface of the glass leads to bone bonding (Rezwan et al. 2006; Hench et al. 1971; Hench 1998, 1999). The bone-bonding mechanism of bioactive glasses has been proposed by Hench, as demonstrated in Fig. 1.

An added advantage of bioactive glasses is that ionic dissolution products from the reactions on bioactive glasses’ surfaces can induce intracellular and extracellular response, stimulating new bone formation (osteogenesis) (Xynos et al. 2001; Sun et al. 2007). There are also studies showing that 45S5 Bioglass^®^ can enhance the secretion of vascular endothelial growth factor (VEGF) and VEGF gene expression in vitro, as well as vascularisation in vivo (Day et al. 2004). Given all these remarkable advantages of 45S5 Bioglass^®^, it makes a sense that 45S5 Bioglass^®^ has been used in a number of commercial products for treatment of bones, joints and teeth. For example, NovaMin (GlaxoSmithKline, United Kingdome) in the form of toothpaste has been used to reduce tooth sensitivity. NovaBone (Alachua, Florida) as a bone-filler material has been used for the treatment of periodontal disease. The latter has also exhibited good performance as an autograft in posterior spinal fusion operations during a period of a 4-year follow-up study, with fewer infections (Jones 2013).

While the application of bioactive glasses in biomedical implants in the past 20 years has demonstrated their excellent performance, the problems associated with their high brittleness and low fracture toughness remain to be addressed (Table 4). To overcome these problems, the composites between bioactive glasses and polymers are needed (Chen et al. 2008; Rezwan et al. 2006; Chen et al. 2012; Roether et al. 2002; Lu et al. 2003; Zhang et al. 2004).

A general issue with bioceramics is that mechanical strength and biodegradability, which are two essential requirements of bone tissue scaffolds, are antagonistic to each other. Mechanically strong materials (e.g. crystalline HA and related calcium phosphates) are virtually bioinert, and biodegradable materials (e.g. bioactive glasses) tend to be fragile. Sintering Na_2_O-containing bioactive glasses into a mechanically capable glass ceramics or fully crystalline ceramics has been proven to be a strategy to achieve mechanical strength competence while retaining good biodegradability in the material (Chen et al. 2006).

### Biocomposites

To mimic natural bone, the composites of polymers and ceramics (biocomposite materials) have been studied and developed in an attempt to increase both the mechanical and biological performances of the scaffolding materials (Mano et al. 2004). Taking advantage of the polymers’ toughness and the ceramics’ strength, their composite materials could have a satisfactory combination of both properties. Moreover, the addition of bioactive ceramic phases to polymer phases will not only counteract the poor bioactivity of polymers, but also buffer the acidic degradation products of polymers (Niemelä and Kellomäki 2011; Shokrollahi et al. 2010).

#### Polymer/calcium phosphate composites

For over three decades, calcium phosphate ceramics such as HA and β-TCP have been used as bone substitutes. However, their application alone is limited due to the difficulty in the fabrication of highly porous structures and their mechanical brittleness. Polymer/calcium phosphate composites fabricated by the addition of a calcium phosphate ceramic to the polymer have been demonstrated to have good biocompatibility. Many reviews have been published on the composites of HA or β-TCP and biodegradable polymers in terms of their in vitro and in vivo performances as scaffolds in bone tissue engineering. The study of Laurencin (Attawin et al. 1995; Laurencin et al. 1996; Devin et al. 1996) demonstrated that porous scaffolds made from a PLGA/HA composite enhanced cell proliferation and differentiation, as well as bone mineral formation, compared with the PLGA group. Cao and Kuboyama (2010) reported that PGA/β-TCP composite showed a better osteoconductivity and enhanced new bone formation within 90 days during the repair of critical-sized bone defects in rat femoral medial-epicondyles compared with PGA/HA composite and implant-free controls.

#### Polymer/bioglass composites

In the past two decades, a great deal of progress has been made with bioactive glass/polymer composites. Silicate bioactive glasses are thought to have a future in bone tissue engineering because they exert a genetic control regulation over the osteoblast cycle and rapid expression of genes. Silicon has been found to have an effect on bone mineralisation and gene activation (Xynos et al. 2001; Sun et al. 2007; Day et al. 2004). There has been a great deal of research published on this subject. For example, PLA and bioactive glass composites have been developed. It has been found that the composites could exhibit the formation of calcium phosphate layers on their surfaces and support rapid and abundant growth of human osteoblasts and osteoblast-like cells during in vitro test (Zhang et al. 2004; Blaker et al. 2003, 2005; Boccaaccini et al. 2003; Li and Chang 2004; Lu et al. 2003; Maquet et al. 2003; Maquet et al. 2004; Navarro et al. 2004; Stamboulis et al. 2002; Verrier et al. 2004). Additionally, biodegradable polymer-coated porous Bioglass^®^ composite scaffolds exhibited enhanced strength compared with the bared ceramic scaffolds (Blaker et al. 2005; Chen and Boccaccini 2006; Bretcanu et al. 2007, 2009; Bretcanu and Boccaccini 2012; Metze et al. 2013).

The compressive modulus of a composite scaffold depends not only on the porosity and pore size of the composite scaffold, but also on the content of the ceramic or glass added. It must be mentioned that only a few composite scaffolds presented in Table 5 were found to have the modulus that could reach in the range of the modulus of the cancellous bone. Hence, further development and selection of scaffolding biomaterials for hard tissue support are needed.

**Table 5:** Porous composites scaffold designed for bone tissue engineering (Chen et al. 2008; Rezwan et al. 2006)

Scaffold composite		Percentage of ceramic (wt %)	Porosity (%)	Pore size (μm)	Modulus (MPa)
Ceramic	Polymer				
Amorphous CaP	PLGA	28–75	75	>100	65
HA	PLLA	50	85–96	100 × 300	10–14
	PLGA	60–75	81–91	800–1,800	2–7.5
	PLGA		30–40	110–150	337–1,459
Bioglass^®^	PLGA	75	43	89	51
	PLLA	20–50	77–80	~100	137–260
				~10	
	PLGA	0.1–1		50–300	
	PDLLA	5–29	94	~100	
				10–50	
Cancellous bone				100–500	100–500

### Summary of scaffolding biomaterials

The ideal biomaterial used for tissue engineering should be mechanical capable, bioresorbable, biocompatible and supportive to cell attachment, proliferation and differentiation. In addition, it should degrade at a physiologically relevant rate. This goal has not yet been achieved. To design a new composite scaffold, it is necessary to weigh up the advantages and disadvantages of the potential biomaterials. A comparison of all scaffolding biomaterials (polymeric materials, bioceramics and biocomposites) is provided in Table 6. Among polymeric materials, amorphous PDLLA is one of the most interesting scaffolding polymers as a coating material in orthopaedic applications because it shows excellent biocompatibility in vivo, good osteoconductivity and high mechanical stability (Schmidmaier et al. 2001a, b; Gollwitzer et al. 2005). Moreover, low-molecular weight PDLLA coating can be used to deliver drugs such as growth factors, antibiotics or thrombin inhibitors (Schmidmaier et al. 2001; Gollwitzer et al. 2003). Cross-linked synthetic polyester elastomer, particularly PGS, has also attracted a great deal of attention for use as scaffolding biomaterials because it is able to provide mechanical stability and structural integrity to tissues or organs without mechanical irritation to the host tissues or organs. Importantly, it has the potential to be tailored in the degradation rates to match clinical requirements.

**Table 6:** Advantages and disadvantages of different scaffolding biomaterials in bone tissue engineering (Chen 2007)

Biomaterials	Advantages	Disadvantages
Naturally derived biopolymers: Collagen Chitosan	Low toxicity; Good biocompatibility; Bioactive; Biodegradability	Low mechanical, thermal and chemical stability; Possibility of immunogenic response
Synthetic polymers Poly(lactic acid) Poly(glycolic acid) Poly(caprolactone) Poly(lactic-*co*-glycolic acid)	Good biocompatibility; Biodegradability; Bioresorbability; Good processability; Good ductility	Inflammatory caused by acid degradation products; Limited mechanical property; Slow biodegradability
Synthetic elastomers Poly(glycerolsebacate) (chemically crosslinked)	Soft elasticity; Good *in vivo* biocompatibility with mild foreign responses; Tuneable degradability	Degrade too fast; Mild cytotoxicity
Calcium phosphates (e.g. HA, TCP and related calcium phosphate)	Excellent biocompatibility; Supporting cell activity; Good osteoconductivity;	Brittle; Slow biodegradation in the crystalline phase
Bioactive silicate glasses	Excellent biocompatibility; Supporting cell activity; Good osteoconductivity; Vascularisation; Rapid gene expression; Tailorable degradation rate	Brittle and weak
Composites (containing bioactive phases)	Excellent biocompatibility; Supporting cell activity; Good osteoconductivity; Tailorable degradation rate; Improved mechanical properties	Still not as good as natural bone matrix; Complex fabrication

Among the bioactive ceramics and glasses shown in Table 6, bioactive silicate glasses offer great opportunities to enhance vascularisation, exert the rapid expression of genes, and tailor their degradation rate. The controllable biodegradability of bioactive glasses makes them advantageous over HA and related CaP. For these reasons, 45S5 bioactive glass is the material of choice for this project. Although bioactive glasses are brittle with low fracture toughness (Table 4), the composites of these materials with polymers can alleviate these disadvantages.

## Scaffolding techniques

### Design parameters of scaffolds for bone engineering scaffolds

In an organ, cells and their ECM are usually organised into 3D tissues. Therefore, in tissue engineering, a highly porous 3D matrix (scaffold) is often necessary to accommodate cells and to guide their growth and tissue regeneration in three dimensions. The structure of bone tissue varies with its location in the body. Hence, the selection of configurations, as well as appropriate biomaterials, will depend on the anatomic site for regeneration, the mechanical loads present at the site, and the desired rate of incorporation. First, the matrix should have a high porosity and a proper pore size to support cell migration, new tissue deposition, and nutrient delivery. Second, the anatomically shaped matrix should be designed to guide new bone formation. Third, the rate of degradation should match the healing rate of the new tissue, should be neither too fast nor too slow (probably 6 months for in vivo applications) (Temenoff et al. 2000). The most important parameters of bone-scaffold design are listed in Table 7.

**Table 7:** Scaffold design parameters for bone tissue engineering application (Temenoff et al. 2000)

Parameters	Requirement
Porosity	Maximum without compromising mechanical properties significantly
Pore size	300–500 $\upmu$m
Pore structure	Highly interconnected
Mechanical properties	
Cancellous bone	Tension and compression
	Strength: 5–10 MPa
	Modulus: 50–100 MPa
Cortical bone	Tension
	Strength: 80–150 MPa Modulus: 17–20 GPa
	Compression
	Strength: 130–220 MPa
	Modulus: 17–20 GPa
	Fracture toughness: 6–8 MPa $\sqrt m$
Derivative properties	
Degradation time	Must be tailored to match the application in patients
Degradation mechanism	Bulk or surface erosion
Biocompatibility	No chronic inflammation
Sterilisability	Sterilisable without altering material properties

### Conventional fabrication techniques of bone scaffolds

**Fig. 2:**
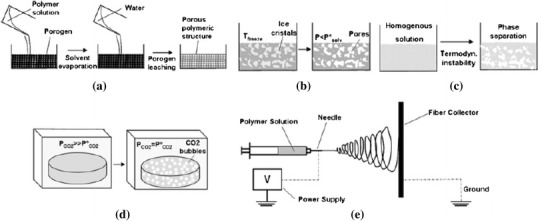
Schematic presentation of commonly used techniques for scaffold fabrication: **a** solvent casting/particulate leaching; **b** freeze-drying; **c** TIPS; **d** gas foaming and supercritical fluid processing; and **e** electrospinning (Puppi et al. 2010)

Numerous methods have been developed and employed to fabricate 3D scaffolds for tissue engineering applications; these can be divided into two principal categories: conventional fabrication techniques (Murphy and Mikos 2007; Morsi et al. 2008; Chen 2011) and solid freeform (SFF) techniques. The latter is also termed ‘rapid prototyping’ (RP) (Chu 2006; Bartolo et al. 2008; Hopkinson and Dickens 2006; Melchels et al. 2012). Each of these techniques produces different features and characteristics of internal architecture, such as pore size, pore structure and interconnectivity, as well as mechanical properties. Therefore, a selection of technology for the scaffold fabrication needs to be made based on a holistic review and comparison of all relevant techniques. This section provides a review on eight conventional approaches that are widely used for producing bone scaffolds (Fig. 2). Computer-aided manufacturing (Andrade et al. 2002) technologies will be reviewed separately in “Solid freeform fabrication (SFF) Techniques”.

#### Solvent casting

Solvent casting involves dissolution of the polymer-ceramic particle mixture in an organic solvent, and casting the solution into a predefined 3D mould. The solvent subsequently evaporates, leaving a scaffold behind. The advantage of this method is that the preparation process is easy and does not require expensive equipment. However, there are two major disadvantages. First, this approach can only form scaffolds of simple shapes (flat sheets and tubes). Second, the residual solvents left in the scaffold material could denature proteins, and thus be harmful to cells and biological tissues.

#### Solvent casting/particulate leaching

This approach involves casting a mixture of polymer solution and porogen particles such as sieved salt or sugar particles, and inorganic granules to fabricate porous membranes or 3D networks (Cao and Kuboyama 2010; Guan and Davies 2004; Hayati et al. 2011). The size of porogen particles and the ratio of polymer to porogen directly control the internal pore size and porosity of the final scaffold, respectively. After solvent evaporation, the dried scaffolds are fractionated in water or a suitable solvent to remove particulates. Once the porogen particles have been completely leached out of the mixture, a porous structure is obtained. This method has both advantages and disadvantages similar to the solvent casting technique.

#### Freeze-drying

This method also requires the use of organic solvents or water to produce a porous scaffold but does not require the use of porogen particles. First, a synthetic polymer is dissolved into a suitable solvent. Subsequently, the solution is poured into moulds of specified dimensions and frozen with liquid nitrogen. The frozen polymer is lyophilised to produce porous scaffolds of highly interconnected pores with porosities being up to 90 %. One of the great benefits of this technique is the ability to fabricate a scaffold without the use of a high temperature. Further, the pore size and the morphology of the scaffolds depend on specific processing parameters, including the freezing rate, temperature and polymer concentrations. However, sponge scaffolds produced by this technique exhibit a porous structure of irregular and small pore size, typically ranging from 15 to 35 μm.

#### TIPS

This approach involves the use of a volatile organic solvent of a low melting point to dissolve the polymer mixed with/without ceramic particles. To induce phase separation, the polymer solution is first cooled rapidly. This leads to the solidification of solvent, which forces the polymer solute into the interstitial spaces. Subsequently, a porous scaffold is obtained after the evaporation of solvent via sublimation. A control of the large number of variables, including types of polymer and solvent, polymer concentration and phase separation temperature allows the generation of a variety of scaffold architectures (Nam and Park 1999; Molladavoodi et al. 2013). The principal advantage of this method is that a high porosity can be achieved by adjusting the parameters. It has been shown that the use of thermally induced phase separation (TIPS) followed by freeze-drying can produce scaffolds of a porosity >95 %. Varying the preparation conditions can also tailor the pore morphologies of scaffolds (Yin et al. 2003; Kim et al. 2004; Barroca et al. 2010). However, the pore size of scaffolds produced by this technique is typically <200 μm (Hutmacher 2000), which limits its utility in bone tissue engineering.

#### Gas foaming/supercritical fluid processing

The high-pressure gas-foaming technique employs a gas as a porogen to create interconnected pores. It was developed to eliminate the use of organic solvents, the residual of which might result in an inflammatory response after implantation. This fabrication process can be conducted at mild conditions. CO_2_, a non-toxic and non-flammable gas, has been widely used in supercritical fluid processing. First, a polymer is placed in a chamber and then saturated with high-pressure CO_2_. As the pressure is rapidly dropped, the nucleation and formation of pores occur as a result of the thermodynamic instability in the gas/polymer system (Mooney et al. 1996). The fabrication parameters such as temperature, pressure, degree of saturate and depressurisation time have a great influence on the pore morphology and pore size of the scaffolds. The gas-foaming technique typically produces a sponge-like structure with the average pore size in the range of 30–700 μm and a porosity up to 85 % (Chen 2011). The drawbacks of this process include the use of the excessive heat during compression moulding; closed, non-interconnected pore structures, and a non-porous skin layer at the surface of the final product.

To achieve a highly interconnected network, a combination of high-pressure gas foaming and particulate leaching techniques is developed. Using this combinatory technique, Harris et al. (1998) have produced PGLA scaffolds of various porosity by adjusting the salt/polymer ratio and salt particle size. The overall porosity of their products was improved up to 97 %.

#### Textile technology (electrospinning)

Electrospinning is a versatile process that involves the use of an electrical charge to create non-woven scaffolds from a polymer solution. This technique allows the fabrication of various fibre patterns with a higher porosity. A number of variables, including solution viscosity, polymer charge density, polymer molecular weight and electric field strength, can be adjusted to control the fibre diameter and morphology (Pham et al. 2006). To date, the electrospinning technique has been widely used to fabricate scaffolds for tissue regeneration applications because it possesses great advantages, including producing fibres with diameters from few microns down to the nanometre range, and highly porous scaffolds with interconnected pores. The disadvantage of this technique is that it involves the use of organic solvents, which could be toxic to cells if not completely removed (Mikos and Temenoff 2000).

#### Powder-forming processes

**Fig. 3:**
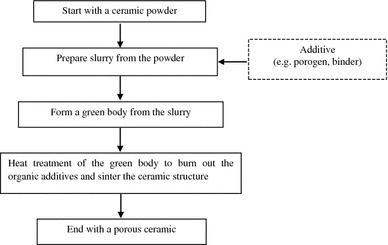
Flowchart of the powder sintering method to produce a porous ceramic scaffold (Chen 2011)

The powder-forming process (Fig. 3) was developed for the fabrication of porous ceramic and glass scaffolds. In this process, a suspension of ceramic particles in a suitable liquid (such as water or ethanol) called slurry is used to prepare green bodies. Fillers such as sucrose, gelatine, PMMA microbeads and a wetting agent (i.e. a surfactant) are added into the ceramic suspension, and these chemicals will produce porosity when they are evaporated or burned out during sintering (Chen 2011). In addition, the presence of binders such as polysaccharides (Haugen et al. 2004), poly(vinyl alcohol) (PVA) (Andrade et al. 2002), and poly(vinyl butyl) (PVB) (Kim et al. 2003) in slurries plays an important role in improving the strength of the green body before the product is sintered (Reed 1988).

The methods for forming green bodies can be classified as dry and wet processes (Ishizaki et al. 1998), as listed in Table 8. Depending on the preparation procedure, each type of method provides a unique geometric shape of ceramic products and porous structure in ceramic.

**Table 8:** Methods of obtaining green bodies for 3D porous ceramics

Processes	References
Dry processes	
1. Loose-packing	
2. Compaction	(Brovarone et al. 2006, 2008; Brown et al. 2008)
Uniaxial-pressing	
Cold-isostatic-pressing (CIP)	
Wet processes	
3. Slip-casting	(Montanaro et al. 1998)
4. Injection-moulding	
5. Phaseseparation/freeze-drying	(Fukasawa et al. 2001)
6. Polymer-replication	(Chen et al. 2006; Schwartzalder and Somers 1963; Chen et al. 2008; Fu et al. 2008; Liu et al. 2009)
7. Gel-casting	(Ramay and Zhang 2003; Potoczek et al. 2009; Wu et al. 2011; Tulliani et al. 2013)

**Fig. 4:**
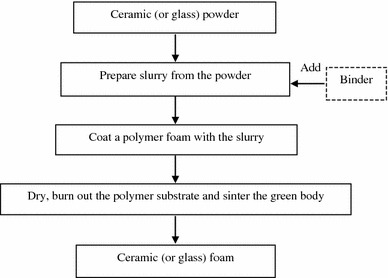
Flowchart of fabrication of ceramic or glass foams via polymer foam replication (Chen 2011)

Among these processes, the replication technique, also named the ‘polymer-sponge’ method (Fig. 4), has gained considerable attention, as it offers the potential of forming uniform dispersion of ceramic powder within a template, resulting in controllable pore size, high porosity and interconnectivity in scaffolds. For this reason, this review highlights the replication technique. In this process, a polymer foam with the desired macrostructure (e.g. polyurethane) is immersed in a ceramic slurry to prepare the green bodies of ceramic foams. After drying, ceramic-coated polymer foam is subsequently heated to decompose the polymer foam, and then the ceramic is sintered to the desired density. Using this technique, Chen et al. (2006) have produced a porous 45S5 Bioglass^®^ scaffold with porosity of ~90 % and pore size ranging from 510 to 720 μm. The sintering conditions have also been optimised to achieve much improved mechanical stability in Bioglass^®^ scaffolds with good bioactivity maintained. In subsequent work, Chen and Boccaccini (2006) successfully toughened their fabricated 45S5 Bioglass^®^ foams by applying a PDLLA coating.

#### Sol–gel techniques

**Fig. 5:**
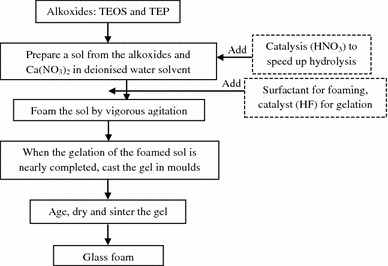
Flowchart of the production of bioactive glass foams using sol–gel process (Chen 2011)

Sol–gel is a versatile process, involving forming a sol by the addition of a surfactant, followed by condensation and gelation reactions (Fig. 5). This technique is based on the chemical reaction of inorganic polymerisation of metal alkoxides. Using the sol–gel process, it is possible to fabricate ceramic or glass materials in a variety of forms, including ultra-fine or spherical-shaped powders, thin-film coatings, ceramic fibres, microporous inorganic membranes, monolithic ceramics and glasses, and highly porous aerogel materials (Chen 2011; Raucci et al. 2010; Chen et al. 2010, 2012; Chen and Thouas 2011; Sepulveda et al. 2002). Despite its advantages, the sol–gel technique does not produce porous ceramics of high mechanical strength. Very recently, the research team led by Chen et al. (2010) successfully developed a sol–gel process of Na_2_O-containing bioactive glass ceramics, which was reported to have improved mechanical strength without losing a satisfactory biodegradability. However, the mechanical properties of the sol–gel-derived 45S5 Bioglass^®^ ceramic scaffolds are not as the same as those of bone.

#### Limitation of conventional fabrication techniques

**Fig. 6:**
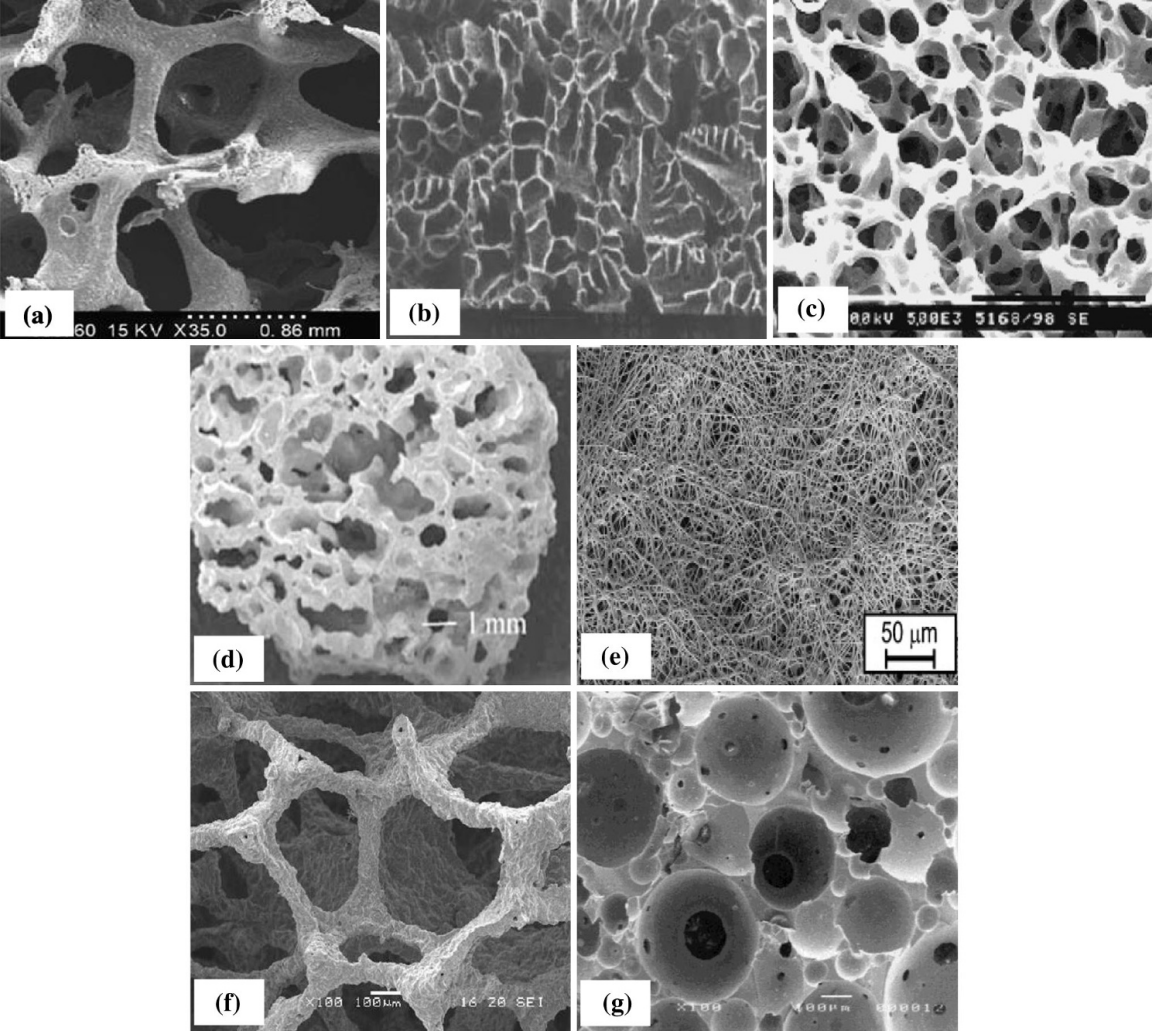
Typical pore morphologies of porous scaffolds by various techniques: **a** solvent casting/particulate leaching (Dalton et al. 2009); **b** freeze-drying (Morsi et al. 2008); **c** TIPS (Dalton et al. 2009); **d** gas foaming (Morsi et al. 2008); **e** electrospinning (Dalton et al. 2009); **f** replication technique (Chen et al. 2008); **g** sol–gel technique (Sepulveda et al. 2002)

Ideally, the scaffold for bone tissue engineering should be porous with appropriate pore size and high interconnectivity to encourage cell penetration, tissue ingrowth and rapid vascular invasion, as well as nutrients delivery. It should also be designed to provide proper mechanical integrity and degrade later at a rate to match the healing kinetics of injured bone. Although the conventional fabrication techniques that have been described have produced scaffolds used in tissue engineering of various types, most of them are incapable of producing fully continuous interconnectivity and uniform pore morphology within a scaffold. Additionally, the pore size, pore geometry and spatial distribution cannot be precisely controlled in these conventional processes. Some conventional techniques are manual-based, with poor reproducibility. Another limitation of most conventional fabrication methods is the need of an organic solvent to dissolve polymers and other chemicals, as well as the use of porogens to create pore structures. Most solvents and porogens are toxic, and their residues in the scaffold may cause severe inflammatory responses. Figure 6 shows the porous morphologies produced by each of these conventional fabrication techniques, and Table 9 provides the details on average pore size, porosity and architecture of the scaffolds produced by these techniques (Hutmacher 2000; Leong et al. 2003).

**Table 9:** Summary of advantages and disadvantages of each conventional technique commonly used in scaffold fabrication (Chen 2011; Hutmacher 2000; Leong et al. 2003)

Technique	Pore size (μm)	Porosity (%)	Architecture	Advantages	Disadvantages
Solvent casting/particulate leaching	30–300	20–50	Spherical pores	Simple method; controlled porosity and pore size	Possibility of residual of solvent and salt particles; structures generally isotropic; insufficient mechanical integrity for use in load-bearing application
Freeze-drying	15–35	>90	High volume of interconnected micropores	Pore structure with high interconnectivity; good porosity	Insufficient mechanical integrity for use in load-bearing application; small pore sizes
Thermally induced phase separation	5–600	<90	High volume of interconnected micropores	Simple method; high porosities; pore structure with high interconnectivity; controllable structure and pore size by varying preparation conditions	Long time to sublime solvent; possibility of solvent residual; shrinkage issues; small scale production
Gas foaming/supercritical fluid processing	30–700	>85	High volume of non-interconnected micropores	Free of toxic solvents; control of porosity	Insufficient mechanical integrity for use in load-bearing application; inadequate pore interconnectivity; possibility of closed pore structure; formation of an outer skin
Textile technology (electrospinning)	<1–10	90		Simple method; high interconnected porosity; high surface area to volume ratio	Insufficient mechanical integrity for use in load-bearing application; possibility of solvent residual; limitation of thickness
Powder-forming processes (bioglass produced by replication technique)	300–700	>80	High volume of interconnected micropores	Simple method; porous structure similar to sponge bone; highly porous and with open pores; free of toxic chemicals	Insufficient mechanical integrity for use in load-bearing application
Sol–gel techniques (bioactive glasses)	>600	>70		High surface area; microstructure similar to that of dry human trabecular bone	Insufficient mechanical integrity for use in load-bearing application; possibility of solvent residual

## Solid freeform fabrication (SFF) techniques

### Overview of SFF techniques

Fabricating a satisfactory biomimetic bone substitute is still a challenge in the field of bone tissue engineering. To control precisely the porous architecture of the scaffold, various SFF techniques, also known as RP, have been developed. In essence, this technology is based on a computer-aided design (CAD) to fabricate custom-made devices directly from computer data. In these techniques, complex scaffold architecture is manufactured in a layer-by-layer manner that builds via the processing of solid sheet, liquid or powder materials stocks according to its computerised cross-sectional 3D image. Unlike the conventional techniques described in “Conventional fabrication techniques of bone scaffolds”, SFF techniques have significant advantages over those conventional techniques in terms of consistency, reproducibility of designed scaffolds and the capabilities of precise control over the architecture of 3D scaffolds such as internal structure, geometry, pore sizes and spatial distribution so that both biological and mechanical performances of tissue-engineered constructs can be improved (Leong et al. 2003; Yeong et al. 2004; Hutmacher et al. 2004).

The brief definitions of technical terms used in the SFF techniques described by Grimm (Grimm, 2004) are listed in alphabetical order as follows:

1. two dimensional (2D): the term indicates that the resulting file is a flat representation with dimensions in only the X and Y axes
2. 3D: abbreviation for three dimensional—the term indicates that the resulting file is a volumetric representation with dimensions in the X, Y, and Z axes
3. accuracy: the difference between an intended final dimension and the actual dimension as determined by a physical measurement of the part in addition to those for linear dimensions, there are accuracy specifications for such features as hole sizes and flatness
4. CAD: a software program for the design and documentation of products in either 2D or 3D space
5. CAM: a software program that uses the design data of CAD to build tool paths and similar manufacturing data for the purposes of machining prototypes, parts, fixtures, or tooling
6. facet: a polygonal element that represents the smallest unit of a 3D mesh
7. feature: discrete attributes of a model or prototype that include intrinsic geometric parameters (i.e. length, width, depth, holes, slots, ribs, bosses, snap fits) and other basic elements of a product design. Figure 7 presents an example of designed unit cell architectures based on different feature primitives.

**Fig. 7:**
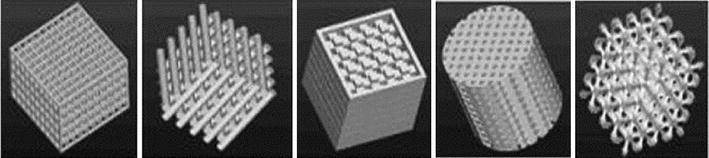
The designed scaffold unit cells based on different feature primitives (Sun et al. 2007)
8. layer thickness: the vertical dimension of a single slice of a stereolithography (SLA) file
9. minimum feature size: the smallest detail of an object that can faithfully be reproduced
10. part finish: a qualitative term for the appearance of a part
11. primitive: simple geometric shapes of a solid model, such as a cube, cylinder, sphere, cone, or pyramid
12. resolution: the minimum increment in dimensions that a system achieves—it is one of the principal determining factors for finish, appearance and accuracy (but certainly not the only one)
13. road, road width, gap width and raster angle: the terms, ‘road’, ‘road width’ and ‘gap width’ are applied to the fused deposition modelling (FDM) process—an illustration of road (many deposited lines of material), road width (diameter of the circular cross-section of the road [measured in *X*–*Y* plane]), gap width (space between roads), raster angle (direction of deposited road) is provided in Fig. 8.

**Fig. 8:**
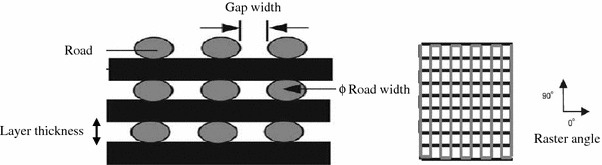
Cross-sectional structure viewed in the *X*–*Z* plane and direction of the FDM-build part (Zein et al. 2002)
14. STL: a neutral file format exported from CAD systems for use as input to RP equipment—the file contains point data for the vertices of the triangular facets that combine to approximate the shape of an object
15. slice: a single layer of an SLA file that becomes the working surface for the additive process
16. support structure: a scaffold of sacrificial material upon which overhanging geometry is built—it is also used to attach rigidly the prototype to the platform; after prototype construction, it is removed in a post-processing operation
17. voxel: a shortened term for volume cell.

**Fig. 9:**
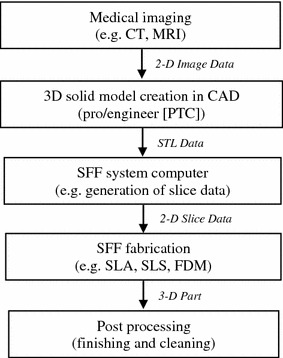
Flowchart presenting typical CAM technology (Leong et al. 2003)

The technological flowchart of all RP techniques is illustrated in Fig. 9.

Among a number of SFF techniques, SLA, selective laser sintering (SLS), laminated object manufacturing (LOM™), ink-jet printing technologies [i.e. 3D printing (3DP)], and FDM are most widely used for the construction of tissue engineering scaffolds. SFF offers a number of great benefits, which are summarised below (Leong et al. 2003):

1. Customised design: using CAD modelling, SFF techniques can manufacture complex scaffolds based on patient-specific data from a medical imaging technique.
2. Computer-controlled fabrication: SFF techniques are able to fabricate scaffolds of highly accurate and consistent pore morphology, using a minimum labour. High porosity (up to 90 %) and full interconnectivity can easily be achieved. These techniques can also reproduce highly complex architectures in a relatively short time without using a mould.
3. Anisotropic scaffold microstructures: SFF techniques can produce macroscopic and microscopic structural features in different regions of the same scaffold; this could lead to the hierarchical structures of multiple cell types (Crouch et al. 2009). With an SFF technique, it is easy to fabricate a functionally graded scaffold (FGS) that has different mechanical properties at different areas of the same scaffold (Chua et al. 2011; Hutmacher et al. 2004).
4. Processing conditions: SFF techniques are flexible because they work under a diverse range of processing conditions, including solvent-free and/or porogen-free processes and mild temperature.

The remainder of this review will focus on the four most frequently used techniques (i.e. SLA, SLS, 3DP and FDM) in the field of tissue engineering.

### SLA

#### Principle of SLA

**Fig. 10:**
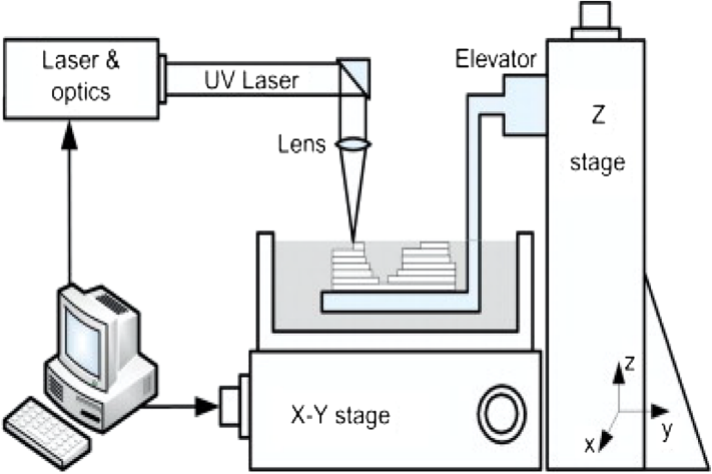
Schematic representation of an SLA system (Chu 2006; Bartolo et al. 2008; Hopkinson and Dickens 2006)

SLA, the oldest of the SFF technologies, was developed by 3D Systems in 1986. It has since been widely used in the field of biomedical engineering. The system of SLA, as demonstrated in Fig. 10, consists of a tank of photo-sensitive liquid resin, a moveable built platform, an ultraviolet (UV) laser to irradiate the resin, and a dynamic mirror system. The SLA process employs a UV laser to build a photo-sensitive liquid resin material layer-by-layer into a 3D scaffold. Once one layer is completely solidified onto a platform, the platform is vertically lowered with a small distance into the resin-filled vat. Subsequently, an amount of liquid resin covers the previous layer, forming the next layer. These steps are repeated until a complete 3D part is formed. Finally, uncured resin is washed off and the scaffold is post-cured under UV light, yielding a fully cured part (Chu 2006; Bartolo et al. 2008; Hopkinson and Dickens 2006).

#### SLA-produced scaffolds used in tissue engineering

**Fig. 11:**
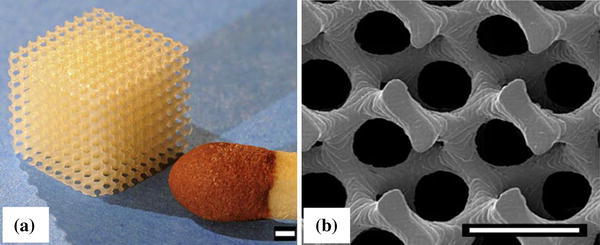
Images of PDLLA scaffolds built by SLA. **a** Photograph; and **b** SEM micrograph (*scale bars* represent 500 μm) (Melchels et al. 2009)

SLA can fabricate 3D scaffolds from polymers, bioceramics and composites. The spatial resolution is usually approximately 50 μm. SLA has been applied to biodegradable polymers, such as poly(propylene fumarate) (PPF) (Cooke et al. 2002; Lee et al. 2007), photocrosslinkable PCL (Elomaa et al. 2011), PDLLA (Melchels et al. 2009; Jansen et al. 2009) (Fig. 11), vinyl esters (Heller et al. 2009) and photocrosslinkable poly(ester anhydride) (Seppala et al. 2011), to create well-defined scaffolds with interconnected porosity of 70–90 %. Using SLA, Lee et al. (2007) have successfully fabricated highly complex bone scaffolds from PPF and diethyl fumarate (Shuai et al. 2013) resins. In another study, Elomaa et al. (2011) fabricated PCL scaffolds using SLA, showing a highly porous interconnected network with porosity of 70 %, and pore size of 465 μm, with no observable material shrinkage.

The SLA system can also fabricate hydrogel polymer scaffolds. The main difficulty in scaffold fabrication using hydrogel is the development of water-soluble components that are functional and photo-labile (Fisher et al. 2001). Seck et al. (2010) have produced 3D biodegradable hydrogel scaffolds from an aqueous photo-sensitive resin-based methacrylate-functionalised poly(ethylene glycol) (PEG)/PDLLA macromers, using the SLA process. Their scaffolds have a well-defined porous network structure, narrow pore size distribution, and highly interconnected pores.

The research team of Arcaute et al. (2010) has developed 3D PEG-based multi-material scaffolds using SLA. The scaffold is aimed at the micro-scale characteristics that could build a cellular microenvironment with a spatially controlled bioactivity. However, the scaffold is deemed of little use for tissue engineering applications due to the poor shape of the fabricated samples.

SLA is also used to build a ceramic network using a photo-sensitive polymer as a binder. The use of SLA to fabricate bioceramic scaffolds was first explored by Chu et al. (1996, 1997, 2002). A suspension of HA and a low viscosity acrylate resin was printed to form scaffolds, and the resin was subsequently burned out, leaving behind a ceramic scaffold of 50 % porosity. However, to print ceramic-based scaffolds, a ceramic suspension should have a solid content in the range of 20–50 volume percentage in the resin (Stuecker et al. 2003). However, the ceramic suspension at this content level has a very high viscosity, which introduces difficulties to SLA processing. Some researchers have developed an indirect fabrication process to produce bioceramic scaffolds from calcium phosphate (Hollister 2005) and Bioglass^®^ (Padilla et al. 2006; Li et al. 2013) by combining SLA and the casting method. In this indirect process, an epoxy mould is first created by SLA, and then a suspension of ceramic acrylate is cast into the mould. When the mould is removed by thermal treatment, the 3D scaffold with the inverse shape of the mould is obtained.

SLA has also been used for the fabrication of polymer/ceramic composite scaffold. It is often more difficult to process a composite than a polymer due to the high viscosity of polymer/ceramic suspension, which is a result of the addition of the ceramic powder (Melchels et al. 2010). Therefore, SLA has not been used widely for fabricate polymer/ceramic composite scaffolds. Using SLA, Elomaa et al. (2013) successfully produced bioactive glass/methacrylated PCL composite scaffolds with well-defined porosity. The scaffolds were reported to show no unwanted polymer layer covering the Bioglass^®^ particles and were thus able to enhance the attachment and proliferation of human fibroblast.

#### Advanced SLA technology

With the reduction in the laser power and improvement of both lateral and vertical resolutions, new generations of SLA technology have emerged. There are three new technologies micro-stereolithography (μSLA), two-photon polymerisation (TPP), and digital light processing (DLP).

##### μSLA

μSLA has been developed for the fabrication of 3D microstructures in a better resolution. This process employs a single photon beam that can be focused more precisely with a reduced spot size of laser. μSLA fabricates complex 3D micro-scale structures with a layer thickness of less than 10 μm. In a PPF scaffold fabricated by the μSLA process (Lee et al. 2008), the rectangular pore sizes are 250–260 μm, and pores are interconnected in the three dominant directions. The mechanical properties of the PPE scaffold were similar to those of human trabecular bone. Similar work was also reported by Choi et al. (2009), who produced PPF-based 3D scaffolds with interconnected pores of 100 μm in size using a scanning μSLA system. Although there are limitations associated with material shrinkage, overcure of the downward surface and the inability to remove uncured resin, this system plays a role in producing 3D micro-scaffolds for tissue engineering.

**Fig. 12:**
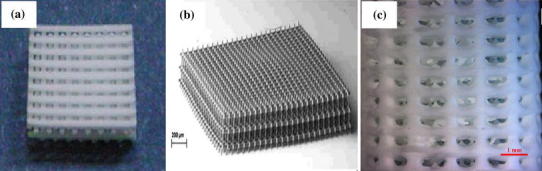
Examples of bioceramics scaffolds built by advanced SLA: structures prepared from **a** HA and TCP using μSLA system (Seol et al. 2013); **b** methacrylated oligolactones using a TPP system (Weiss et al. 2011); and **c** 45S5 Bioglass^®^ using DLP system (Tesavibul et al. 2012)

μSLA has also been used to produce robust ceramic scaffolds from HA and tricalcium phosphate (TCP) (Fig. 12a) by Seol et al. (2013). A slurry was prepared from a HA/TCP powder and photo-curable resin at 20 % volume, and was printed to build a designed 3D structure. The green body was then sintered to remove the resin. The 3D HA/TCP scaffolds have completely interconnected pore sizing around 300 μm. The compressive strength of the above ceramic scaffolds is in the range of human cancellous bone, and the scaffolds are reported to support cell proliferation and osteogenic differentiation.

##### TPP

The development of a TPP system is aimed at fabricating scaffolds at a greater depth, higher resolution up to nanolevel, and an ultra-fast speed. In TPP, when a near-infrared ultra-short-pulsed laser is closely focused into a volume of photo-curable resins, real 3D microstructures can be fabricated using a layer-by-layer accumulating technique, making it a promising technique for 3D nano/microfabrication. In addition, a spatial resolution of sub-100 nm scale has been achieved with TPP by employing a radical quenching mechanism (Melchels et al. 2010; Lee et al. 2008). Using the TPP system, Weiss et al. (2009) produced the first 3D micro-architectures and nano-architectures for cartilage-tissue engineering with a spatial resolution lower than 1 μm. In in vitro investigation using bovine chondrocytes, TPP-structured scaffolds (Fig. 12b) also showed high cytocompatibility as reported by the same group (Weiss et al. 2011).

##### DLP

DLP employs visible blue light. It was based on lithography-based additive manufacturing technologies (AMT), for building ceramic or glass parts. In the DLP process, dynamic masks are used to cure a whole layer at a time. Hence, this technique offers a significantly higher building speed. Other advantages of DLP include a high lateral resolution of 40 μm (~50 μm of conventional SLA), an efficient process for filling a large amount of ceramic particles (~40–60 % solid loading), and no need for expensive specialised equipment such as a laser or a heating chamber (Felzmann et al. 2012). Using the DLP-based process, Felzmann et al. (2012) have produced ceramic scaffolds from 45S5 Bioglass^®^, β-TCP or alumina. Their scaffolds show interconnected pores of 300 μm in size. After sintering, few microcracks were observed in the scaffold material and shrinkage was 20 %. The same group (Tesavibul et al. 2012) also use this technique to fabricate a Bioglass^®^ based porous network (Fig. 12c) as an orthopaedic implant for the maxillofacial area.

#### Advantages and disadvantages of the SLA process

SLA technology is a versatile process that allows the freedom of designing structures, the ability to build parts of various sizes from submicron to decimetre, and a good surface finish. Compared with other SFF techniques, SLA shows excellent reproducibility, producing nearly identical built architectures. This indicates the very high accuracy and resolution of this technique (Heller et al. 2009; Melchels et al. 2010). The porous network architecture produced by SLA is characterised by a much more homogeneous cell distribution compared with that produced by the salt-leaching technique, and allowing more efficient supply of oxygen and nutrients during cell culturing (Melchels et al. 2010).

Nonetheless, the use of photo-sensitive material is primarily considered a limitation of this process. Another disadvantage of this process is associated with the shrinkage of the polymer due to polymerisation. Toxicity such as skin irritation and cytotoxicity caused by photo-sensitive resins also appears to be a major problem. Most recently, resins based on vinyl esters, an alternative resin that possesses better biocompatibility in vivo, have been explored (Heller et al. 2009).

### SLS

#### Principle of SLS

**Fig. 13:**
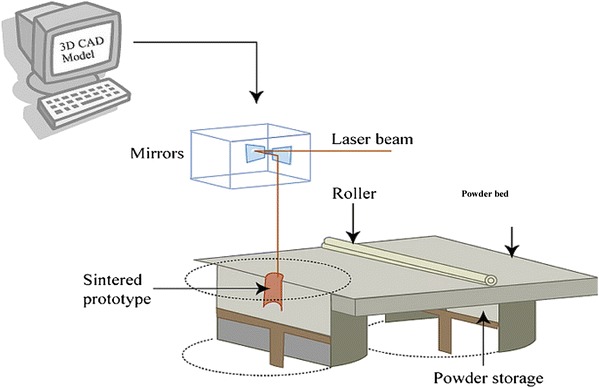
Schematic representation of the SLS system (Chu 2006; Bartolo et al. 2008; Hopkinson and Dickens 2006)

The SLS technique was developed at the University of Texas in Austin in 1986 and was commercialised by DTM Corporation in 1992. It employs a CO_2_ laser beam to fuse (or sinter) selected regions of material powders onto a powder bed surface, forming a material layer. Once a first layer is solidified, the powder bed is lowered by one-layer thickness. The next layer of the material is laid down on the top of the bed by a roller. The process is repeated until the part is completed. The solid powder acts as a structural support, and the residual powder of the sample is removed. An illustration of SLS is shown in Fig. 13 (Chu 2006; Bartolo et al. 2008; Hopkinson and Dickens 2006).

#### SLS scaffolds for tissue engineering

SLS has been used to produce tissue-engineered constructs from polymers, metals and ceramics, especially from biodegradable polymers (Williams et al. 2005; Yeong et al. 2010; Eshraghi and Das 2010; Pereira et al. 2012). Using the SLS technique, Eshraghi and Das (2010) have produced PCL scaffolds with orthogonal porous channels for implants at load-bearing sites. Under optimal fabrication conditions, the PCL scaffold demonstrated accurate dimensions (within 3–8 %) compared with the designed dimensions, nearly full density (>95 %) in the solid struts, and remarkable compressive strength, which is the highest compared with other scaffolds produced by SLS. In another work, P3HB porous network produced by Pereira et al. (2012) with SLS showed accurate geometrical and dimensional features, nearly identical to the virtual model.

Fabricating bioceramic with the SLS technique directly has proven difficult, primarily due to the fast heating and cooling rates associated with the high-energy laser used (Kruth et al. 2003; Lorrison et al. 2005; Cruz et al. 2005). However, an indirect SLS method seems likely to be more feasible for the fabrication of porous scaffolds as reported by Lee et al. (2004; Lee and Barlow 1993; Goodridge 2004). In their studies, bioceramic powder particles were coated with a polymer binder. During the SLS process, the binder layer was melted, and the powder particles were bonded together. In the subsequent sintering process, the binder was burned off and bioceramics were sintered. The scaffolds produced by the SLS technique demonstrated good surface qualities and structural integrity, with flexural strengths at 16 MPa, which is in the range of those of cancellous bone (Goodridge et al. 2007).

In the SLS process, the particle size of the feedstock powder and the content of binder have a critical influence on the mechanical properties of the final scaffold product. In their systematic studies, Kolan et al. (2012) tested the effects of different particle sizes of the feedstock powder and binder content on the quality of bioactive glass porous scaffolds. The compressive strength values of their bioactive glass products range from 41 MPa for a scaffold to 157 MPa for a dense part. The compressive strength of bioactive glass scaffolds decreased 38 % after a six-week incubation in SBF. However, the value was still higher than that of a human trabecular bone, which suggested that the scaffolds may be suitable for load-bearing sites.

**Fig. 14:**
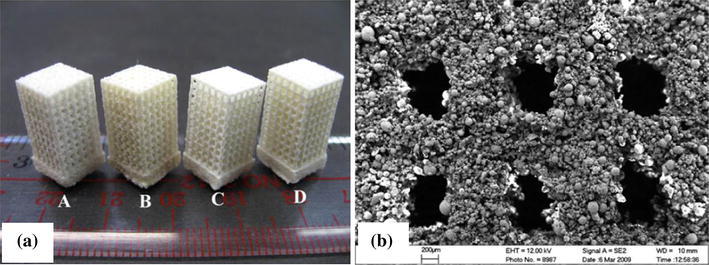
Images of PHBV/TCP composite scaffolds built by SLS: **a** photograph; and **b** SEM morphology (Duan et al. 2010)

The use of SLS has been expanded to polymer/ceramic composites. The major challenge in the fabrication of porous composite scaffolds using SLS is associated with finding an optimal combination of the process parameters, including powder composition, part particle size, laser power, powder bed temperature, scan speed, scan spacing, and part orientation that critically influence the mechanical properties of the scaffolds. The most tested composite system by the SLS process is PCL/HA (Wiria et al. 2007; Eosoly et al. 2010; 2012), PCL/TCP (Lohfeld et al. 2012) and poly(hydroxybutyrate-*co*-hydroxyvalerate) (PHBV)/TCP (Duan et al. 2010; Duan and Wang 2010) (Fig. 14). By optimising the laser power and the scan speed, Wiria et al. (2007) were able to produce a PCL/HA composite scaffold with 10, 20 and 30 wt % of HA. The compressive Young’s modulus of these scaffolds was 34, 24, and 57 MPa, respectively.

The mechanical and biological performances of scaffolds in vivo are greatly influenced by their micro-architectures. Lohfeld et al. (2012) produced PCL/TCP scaffolds with a range of micro-architectures and compositions using the SLS technique. In their work, scaffold fabrication from the composite of up to 50 wt % TCP is demonstrated to be possible. With increasing porosity, the stiffness of the scaffolds is seen to drop; however, the stiffness can be increased by geometrical changes such as the addition of a cage around the scaffolds, especially for small scaffolds. However, the in vivo evaluation showed that the performance of their scaffolds was not as good as the TCP control in new bone formation.

Functional gradient scaffolds that mimic the anatomical geometry of bone have also been produced from PCL using the SLS technique combined with the CAD design (Chua et al. 2011). The porosity and compressive stiffness and yield strengths of their PCL scaffolds are 40–84 %, 3–56 MPa and 0.2–5 MPa, respectively, which are comparable to those of cancellous bone in the maxillofacial region (Sudarmadji et al. 2011).

It is technically difficult to incorporate bioactive molecules in the scaffolds produced by the SLS technique due to the high temperatures used for melting the powders. Using the SLS technique, Duan and Wang (2010) fabricated BSA, loaded CaP/PHBV nanocomposite microspheres into 3D porous scaffolds with good dimensional accuracy while retaining the bioactivity of BSA. In addition, protein-loaded microspheres were subjected to the laser sintering process and the bed temperature of the part was chosen to be 35 °C without further preheating to protect the bioactivity of BSA to the maximal extent.

The scaffolds produced by SLS have been assessed in their cell attachment, proliferation, differentiation and formation of bone tissues (Shuai et al. 2013; Zhang et al. 2008; Bael et al. 2013). Zhang et al. (2008) manufactured HA-reinforced polyethylene and polyamide composites produced by the SLS process to investigate the biocompatibility of SLS composites. The results showed good biocompatibility of the SLS composite processed with no adverse effects observed on cell viability and metabolic activity, supporting a normal metabolism and growth pattern for osteoblast.

#### Advanced SLS technology

To minimise heat transfer, Popov et al. (Popov et al. 2004) developed a technique of surface selective laser sintering (SSLS) to fabricate 3D composite scaffolds that are both bioactive and biodegradable. SSLS is different to conventional SLS in terms of using the laser power and laser intensity. In the conventional SLS process, polymer particles absorb infrared radiation (λ = 10.6 µm) and are completely melted. Melted polymer particles are then fused with each other to form a bulk shape. This process involves large volumetric shrinkage. In the SSLS process, a near-infrared laser radiation (λ = 0.97 µm) is used, which is not absorbed by polymer particles at all. For the sintering purpose, polymer particles are coated with carbon. Hence, the melting of polymer is limited to the surface layer of polymer particles. Since there is no overheating in the particles’ internal region, the SSLS technology has the potential to maintain the nature of delicate biomolecules inside the polymer particles during the scaffold fabrication (Bartolo et al. 2008; Antonov et al. 2005; Kanczler et al. 2009).

#### Advantages and disadvantages of the SLS process

Most steps of the SLS process are similar to those of SLA, but the former enables the processing of powder-based materials by melting or sintering and does not use organic solvents or any toxic chemicals. The SLS technique also eliminates the requirement of an additional supporting structure for the model during processing because unprocessed powders serve as a supporting material. However, SLS has an inherent shortcoming (i.e. heat transfer reactions by radiation, convection and conduction in the feeders and in the powder bed) and as a result, the biodegradable polymer powder is likely to degrade (Pham et al. 2008). Nevertheless, the investigation by Pereira et al. (2012) on both processed P3HB powder and unprocessed P3HB powder has clearly shown that there were no significant differences between the two groups of P3HB in thermal values and chemical shift peaks obtained from DSC and ^1^H-NMR, respectively, indicating that the P3HB powder which underwent printing sets can be re-utilised to print additional structures without affecting the reproducibility of the process. Although the heat generated by the laser beam may not affect the material property at a short time, SLS processing of complex-shaped prototypes or large prototypes typically needs enough time to expose polymers to a high temperature. Another problem of this technique is associated with the almost-impossible removal of powder trapped inside the small hole, which may block cellular ingrowth and induce an adverse inflammatory reaction. Similar to SLA, the shrinkage of the parts during melting or sintering is another principal problem.

### 3D printing (3DP)

#### Principle of 3DP

**Fig. 15:**
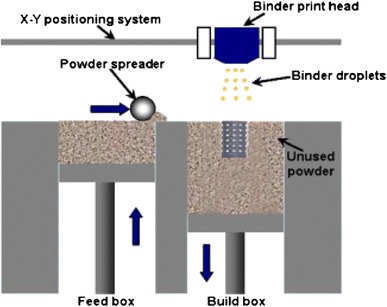
Schematic representation of the 3DP system (Fielding et al. 2012)

3DP is one of the ink-jet printing techniques that was developed at the Massachusetts Institute of Technology (MIT) in 1989. It is employed to create a complex 3D solid object by selective spraying a liquid binder onto the layer of the powder bed; this merges particles together to form a solid layer. The powder bed is then lowered so that a new powder layer is spread over the surface of the previous layer by the roller. This process is repeated until the pre-designed object, which is embedded inside unfused powders, is obtained. The completed object requires the removal of the loose powder. The machine diagram of a 3DP is given in Fig. 15. Subsequently, Therics Incorporation has applied a developed 3DP process named the TheriForm™ process to produce scaffolds for use in tissue engineering (Chu 2006; Bartolo et al. 2008; Hopkinson and Dickens 2006).

#### 3DP applications in tissue engineering

**Fig. 16:**
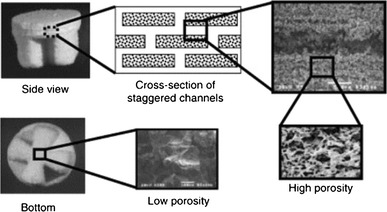
A scaffold with two distinct regions: 90 % porous _D,L-_PLGA/_L-_PLA as the cartilage region (*upper side*) and 55 % porous cloverleaf-shaped _L-_PLGA/TCP as the bone region (*lower side*) (Sherwood et al. 2002)

3DP has been widely used to produce scaffolds from a broad variety of materials, including polymer, hydrogels, ceramics and composites. Currently, most research studies in the 3DP field have focused on evaluating mechanical property and in vitro and in vivo performances. Kim et al. (1998) employed 3DP combined with a particular leaching technique to create a porous PLGA scaffold with an intrinsic network of interconnected channels for a hepatocyte (HC) function study. The pore sizes and porosity of the scaffold were 45–150 μm and 60 %, respectively. Sherwood et al. (Sherwood et al. 2002) reported the fabrication of a device with two distinct regions (cartilage and bone) using the TheriForm™ 3DP process. The upper cartilage region was 90 % porous and composed of _D,L-_PLGA/_L-_PLA. The lower, cloverleaf-shaped bone portion was 55 % porous and consisted of a _L-_PLGA/TCP composite as shown in Fig. 16. The transition region between these two sections contained a gradient of materials and porosity to prevent delamination. In in vitro evaluation, chondrocytes preferentially attached to the cartilage portion of the device, and biochemical and histological analyses showed that cartilage formed during a 6-week culture period.

Ge et al. (2009) reported the fabrication of 3DP-printed PLGA scaffolds and their mechanical properties, microenvironment, and biological properties. The results showed that the PLGA scaffolds examined had mechanical properties (7.8 MPa) similar to that of trabecular bone (7.7 MPa), but was still much weaker compared to cortical bone (193 MPa). In addition, the PLGA scaffolds contain micropores within their macropore walls. Human osteoblasts were found to proliferate upon seeding on the PLGA scaffolds.

3D printing is the only the solid-phase RP technique that is compatible with hydrogel manufacturing. Hydrogels made from PEG/collagen/PDL, PEO/PCL, and starch/cellulose have been processed with the 3DP system for biomedical engineering applications. One major drawback when working with hydrogels is the lack of mechanical strength. Therefore, a post-treatment step involving infiltration and crosslinking with monomers and/or pre-polymer is needed to improve the mechanical stability of the constructs (Billiet et al. 2012). It is possible to incorporate biological agents or even living cells into hydrogel scaffolds because of the involvement of water in hydrogel.

The 3DP system is applicable for the fabrication of ceramic-based tissue engineering scaffolds. However, post-processing with heat treatment is required to achieve higher density and better mechanical properties of the finished parts; this is similar to the SLS process that has been described. Bioceramic powders, such as HA (Roy et al. 2003; Seitz et al. 2005; Warnke et al. 2010), β-TCP (Warnke et al. 2010; Santos et al. 2012) and bioglass (Meszaros et al. 2011), have been fabricated into porous scaffolds by the 3DP system. In one study, Seitz et al. (2005) reported the possibility of using the 3DP process chain to build porous HA scaffolds with internal channels between 450 and 570 μm. The compression strength of the test parts is 21 MPa, falling in the range of those of human spongy and cortical bone. However, the scaffolds were not suitable for carrying high forces in strongly loaded regions in the human skeleton. Likewise, in the research of Santos et al. (2012), differently shaped β-TCP scaffolds were fabricated by the 3DP technique using the patient’s specific CT data. The scaffolds were sintered at different temperatures to enhance their mechanical properties. The porosity, bulk density and compression strength are influenced by parameters such as particle size distribution, sintering temperatures, sintering time length or the binder’s concentration. The results showed a good cell–scaffold interaction.

Another investigation on β-TCP scaffolds was conducted by Fielding et al. (2012). By adjusting the processing parameters of 3DP, scaffolds of a high resolution were produced from TCP powders with or without SiO_2_/ZnO doping. The addition of dopants into the TCP scaffolds was demonstrated to increase the mechanical strength of the scaffolds, as well as cellular proliferation.

**Fig. 17:**
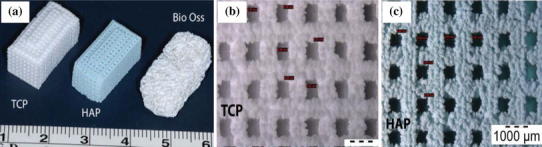
Examples of bioceramic scaffolds produced by 3DP: **a** TCP and HA photograph; **b** SEM image of TCP; and **c** SEM image of HA (Warnke et al. 2010). The magnifications of **b** and **c** are the same

Warnke et al. (2010) investigated the biocompatibility of HA and TCP scaffolds (Fig. 17) produced using a 3DP/sintering technique and their ability to support and promote the proliferation of human osteoblasts compared with the commonly used bone replacement material, bovine HA (BioOss^®^). The results showed that both TCP and HA scaffolds were colonised by human osteoblasts. Cell vitality staining and biocompatibility tests showed superior biocompatibility of HA scaffolds to BioOss^®^, while BioOss^®^ was more compatible than TCP.

3DP has been used for the production of polymer/ceramic composite scaffolds. The composite scaffolds made from PLGA/TCP and 55 wt % salt particles for the bone portion of the osteochondral device were investigated by Sherwood et al. (2002). The tensile strength and compressive strength of the porous PLGA/TCP scaffolds were similar in magnitude to fresh cancellous human bone.

In another investigation, Sharaf et al. (2012) fabricated PCL/TCP (50:50 w/w) composite scaffolds containing channels of either 1 mm or 2 mm in diameter using a TheriForm™ machine, and evaluated porcine bone marrow-derived progenitor cell (pBMPC) proliferation and penetration in the scaffolds. The composite scaffolds with 1-mm channels showed greater cellular proliferation, penetration, and collagen formation after a two-week in vitro cell culture than the scaffolds of 2-mm channels.

Using 3DP, the research team of Parsons et al. (2003; Simon et al. 2003) fabricated PLGA/TCP scaffolds and evaluated the effect of prescribed meso-architecture on bone response in a rabbit model. The results demonstrated that the scaffolds with engineered macroscopic channels and a porosity gradient had higher percentages of new bone area, compared to scaffolds without engineered channels.

Bergmann et al. (2010) employed 3DP process to produce a composite of β-TCP and a bioactive glass similar to the 45S5 Bioglass^®^, using orthophosphoric acid (H_3_PO_4_) and pyrophosphoric acid (H_7_P_2_O_7_) as binders. The maximum resolution (a layer thickness) of the printed structures was 50 μm. In the printing process, the glassy phase of the granules had no effect on the cement reaction. Therefore, the glass content can be varied to generate tailored biodegradation capabilities of the implant. Nevertheless, the blending strength of 15 MPa is still 10 times lower than that of natural bone. Winkel et al. (2012) produced scaffolds from 13 to 93 Bioglass/HA powder using 3DP followed by sintering.

Fierz et al. (2008) studied the effects of different designs and layers of HA scaffolds with tailored pores on osteoblast cell migration and tissue formation. Histological results showed that cells of different morphology could fill the micropores of scaffolds produced by the 3DP technique.

Shanjani et al. (2011) conducted an interesting study in which they investigated the influence of the orientation of the stacked layers on the mechanical behaviour of the 3DP-made calcium polyphosphate (CPP) cylinders. They demonstrated that the scaffolds with layers stacked parallel to the compressive loading direction were about 48 % stronger than those with the layers stacked perpendicular to the loading direction. However, the tensile strength values of the samples were not significantly influenced by the stacking orientation. This study indicates that the compressive strength of the scaffolds can be tailored by the orientation of the powder stacking layers within the CPP structures relative to the loading/stress profile at the implant site.

#### Advantages and disadvantages of 3DP process

3DP is a simple, versatile technique that has several advantages. These include the use of cheap material, the high build speed of the system, and no additional support structure needed during processing similar to the SLS system. In particular, it does not require the use of heat or harsh chemicals, making it friendly for incorporating biologically active molecules inside the scaffolds. However, the final objects have relatively poor strength due to the weak bonds between particles, and the limited resolution and accuracy. Another drawback of the 3DP system is the rough surface finish of objects due to the large size of powder particles. Similar to the problems in the SLS process, it is difficult to remove the powder particles trapped inside small cavities of parts; this may be harmful to cells and tissues. Further, the shrinkage and distortion occur in the both the printing and sintering process.

### Extrusion-based processes

#### Principle of FDM

**Fig. 18:**
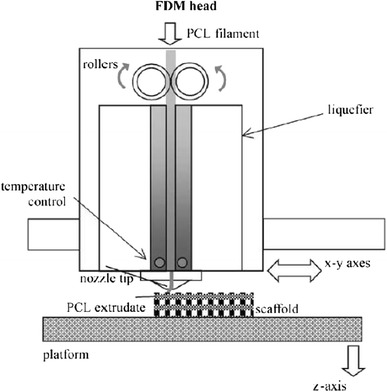
Schematic representation of the FDM system (Zein et al. 2002)

The extrusion-based RP, which is commercially known as the FDM process (Fig. 18), was first developed and commercialised by Stratasys Inc. in 1992. It fabricates 3D scaffolds by melting and extruding material (normally a thermoplastic polymer) through a moveable nozzle with a small orifice onto a substrate platform. The filament material is fed through two rotating rollers into the extruder head, where the material can be melted. The nozzle moves in the *x* and *y* directions so that the filament is deposited on a parallel series of material roads to form a material layer, and subsequently the build platform in the *z* direction is lowered to build the new layer on the top of the first layer. After the extruded material cools, solidifying itself and bonding to the previous layer, a 3D structure is yielded. In general, this technique requires support structures for overhangs or island features. Recently, the FDM system was enhanced to have two nozzles. One nozzle is used for building the material and the second nozzle is used to extrude a different material for the temporary support material. After the part is completed, the support structures would be broken away from the parts (Chu 2006; Bartolo et al. 2008; Hopkinson and Dickens 2006).

#### FDM applications in tissue engineering

The FDM process has been used to produce scaffolds from a wide variety of materials (polymers, ceramics and composites). Zein et al. (2002; Hutmacher et al. 2001, 2008) first used the FDM technique to create PCL honeycomb-like scaffolds (the first generation of scaffolds). PCL scaffolds showed excellent biocompatibility with human fibroblast. The same group later produced PCL scaffolds with honeycomb-like pattern, fully interconnected channel network, and controllable porosity and channel size. The PCL scaffolds were produced with the channel size of 160–170 µm, filament diameter of 260–370 µm and porosity of 48–77 %, and regular honeycomb pores. The compressive stiffness ranged from 4 to 77 MPa, with a yield strength from 0.4 to 3.6 MPa and a yield strain from 4 to 28 %.

Hsu et al. (2007) evaluated PLA and PCL scaffolds produced via FDM for bone and cartilage regeneration. The results indicated that the highly porous and interconnected structure of scaffolds could benefit cell ingrowth. Yen et al. (2009) fabricated PLGA scaffolds using FDM and modified them with type II collagen for cartilage-tissue engineering. The seeded chondrocytes were well distributed inside the hybrid scaffolds with a large spacing of fibre stacking facilitating the removal of acidic degradation products, and neo-cartilage tissue was populated in the scaffolds. Using the FDM process, Tellis et al. (2008) produced poly(butylene terephthalate) (PBT) trabecular scaffolds with various pore structures.

The manufacture of bioceramic scaffolds with the FDM technique can be subdivided into two processes: the fused deposition of ceramics (FDC) and the lost mould technique (Leong et al. 2003; Smay and Lewis 2012). The former is a direct printing technique; the latter is an indirect method. The FDC technique was developed by Cornejo et al. (2000). It uses filament as a precursor to fabricate 3D green ceramic parts. The filament is a composite of thermoplastic polymer, ceramic powder and binder. The thermoplastic polymer and binder are removed during post-processing, and the sintering of the finished ceramic parts is conducted to improve their mechanical properties. The FDC process can be utilised to create ceramic components for scaffolding and bone tissue engineering applications (Danforth et al. 1998; Onagoruwa et al. 2001; Iyer et al. 2008). The lost mould technique uses an FDM machine to produce polymer moulds with a negative structure of the intended network, and then the ceramic slurry is cast into the mould. Once the ceramic slurry solidifies, the finished object will be heated to remove the polymer mould, followed by a sintering process to consolidate the ceramic structure (Bose et al. 1999; Hattiangadi and Bandyopadhyay 2000; Kalita et al. 2003; Bernardo 2010).

**Fig. 19:**
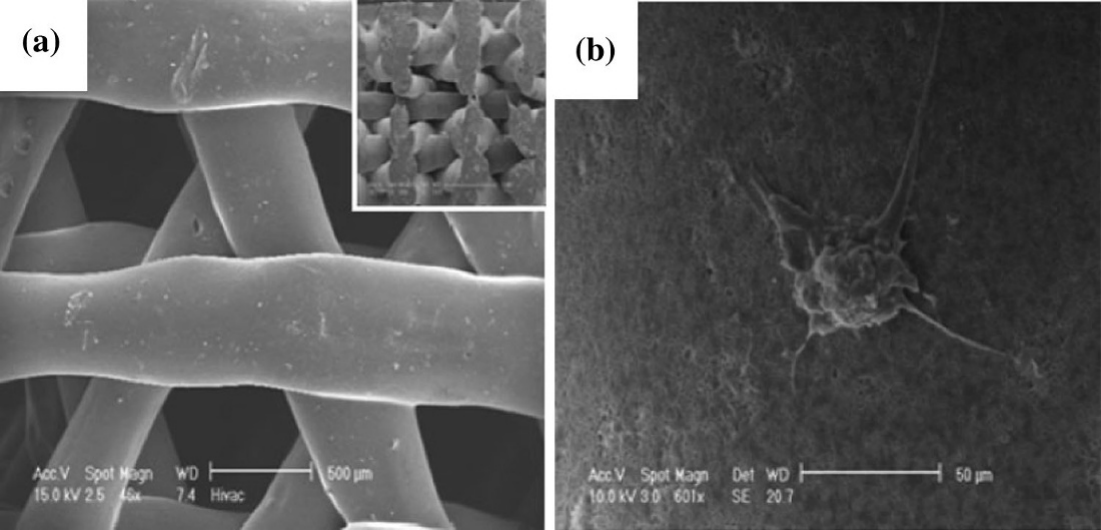
SEM images of PCL/TCP composite scaffolds obtained from FDM: **a** structure of top view with *inset* of cross-sectional view; and **b** osteoblast cells attached on the scaffold surface (Zhou et al. 2007)

The FDM process has been used to produce composite scaffolds. Hutmacher et al. (2008; Hutmacher and Cool 2007) produced the first generation of FDM scaffolds (e.g. PCL/HA and PCL/TCP) for bone tissue regeneration (Fig. 19). The same group has developed the second generation of scaffolds from different polymers and CaP. These composite scaffolds exhibited favourable mechanical properties, degradation and resorption kinetics and bioactivity. In addition, these scaffolds demonstrated improved cell seeding, and enhanced incorporation and immobilisation of growth factors. Recently, Bioglass^®^/polymer composite scaffolds produced via the FDM process were reported by Korpela et al. (2013). Porous scaffolds were created using PLA, PCL, and PCL with S53P4 bioactive glasses.

Lam et al. (2009) investigated the in vivo performance of PCL scaffolds in a rabbit model for up to 6 months. Histological examination of the in vivo samples revealed good biocompatibility, with no adverse host tissue reaction up to 6 months. Zhou et al. (2007) studied the use of mineralised cell sheets in combination with fully interconnected composite scaffolds (PCL/CaP). The scaffolds were implanted subcutaneously in nude rats. Histological and immune histochemical examination revealed that neo-mineralised tissue formed in the constructs and bone formation followed an endochondral pathway.

The quality of the FDM-build scaffold generally depends on the road size, the shape, the uniformity and road consistency. Efforts have been invested to optimise the processing parameters and improve FDM processing efficiency. Anitha et al. (2001) assessed the effect of parameters such as layer thickness, road width and speed deposition on the quality of the prototypes using the Taguchi technique. Recently, Ramanath et al. (2008) made progress in understanding the melt flow behaviour (MFB) of PCL, which was used as a representative biomaterial. The MFB significantly affects the quality of the scaffold; this depends not only on FDM processing parameters but also on the physical properties of the materials used.

#### Advantages and disadvantages of the FDM process

The advantages of this technique include its low cost, the lack of use of organic solvent, the ability to form a fully interconnected pore network in complex 3D architecture, and rare or no requirement of cleaning up the finished objects. This technique allows a flexible fabrication of interconnected porous scaffolds with compositional or morphological variation across the entire matrix, with architecture being highly reproducible. Nonetheless, there are inherent limitations of raw material selection, which needs to be used in the form of filaments with specific size. Other limitations include the effect of high temperatures on raw material, and the lack of adequate resolution. In addition, it is limited in the *z* direction due to the diameter of the extruded filament (Chen et al. 2007). Hence, researchers try to modify FDM to overcome these problems with an attempt to avoid requiring precursor filaments or operating harsh temperatures, as discussed below.

#### Advanced FDM technology

Several modified FDM processes have been proposed for scaffold fabrication. These include multi-head deposition system (MHDS), low-temperature deposition manufacturing (LDM), precision extruding deposition (PED), pressure-assisted microsyringe (PAM), robocasting, and 3D-Bioplotter^®^ system (Yeong et al. 2004; Hutmacher et al. 2008; Hoque et al. 2011).

##### MHDS

**Fig. 20:**
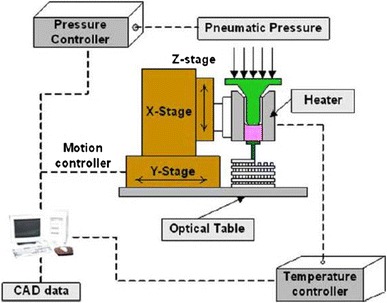
Schematic representation of MHDS (Kim and Cho 2009)

The MHDS (Fig. 20) involves incorporating more than one independent extrusion head into the system to create a complex composition and geometry of scaffolds from various biomaterials. This system can fabricate 3D microstructures with a resolution of several tens of microns. Kim and Cho (2009) fabricated scaffolds from various biomaterials (e.g. PLGA, PCL and TCP) using MHDS. In their work, the deposition process was optimised to achieve efficiently a uniform line width, line height and porosity. Blended 3D PCL/PLGA scaffolds were fabricated with a fully interconnected architecture and a porosity of approximately 70 %. The compressive strength and modulus of the scaffold is approximately 0.8 and 12.9 MPa, respectively (Kim and Cho 2009).

**Fig. 21:**
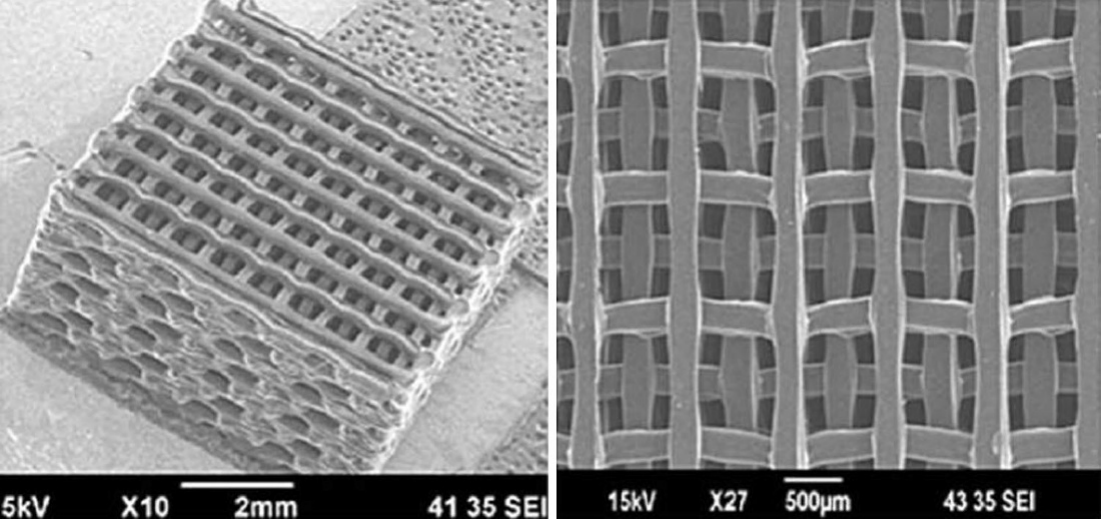
SEM images of PCL/PLGA scaffold fabricated via MHDS (Lee et al. 2012)

The same research group evaluated the PCL/PLGA/TCP scaffolds using osteoblasts. *In vivo* study using the calvaria defect model in rats indicated that scaffolds had the potential to enhance bone formation at 8- and 12-weeks implantation (Kim et al. 2010). Later, Lee et al. (2012) also employed MHDS in the production of PCL/PLGA scaffolds (Fig. 21) with different pore architectures (lattice, stagger, and triangle types) and stacking directions (horizontal and vertical). They found that the mechanical properties of the triangle-type scaffold were the strongest among the experimental groups. Stacking direction affected the mechanical properties of the scaffolds.

A key benefit of using MHDS is the ability to fabricate scaffolds that accommodate different materials in a single layer. Nonetheless, using MHDS involves processing at a high temperature, which leads to the decomposition of polymer materials. In addition, this technique does not allow for cell-loaded or drug-loaded scaffold fabrication.

##### LDM

**Fig. 22:**
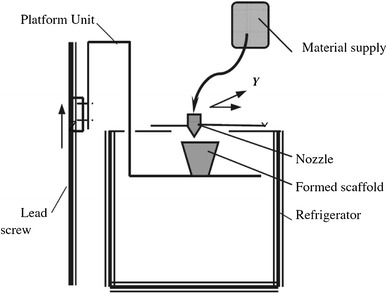
Schematic representation of LDM (Xiong et al. 2002)

**Fig. 23:**
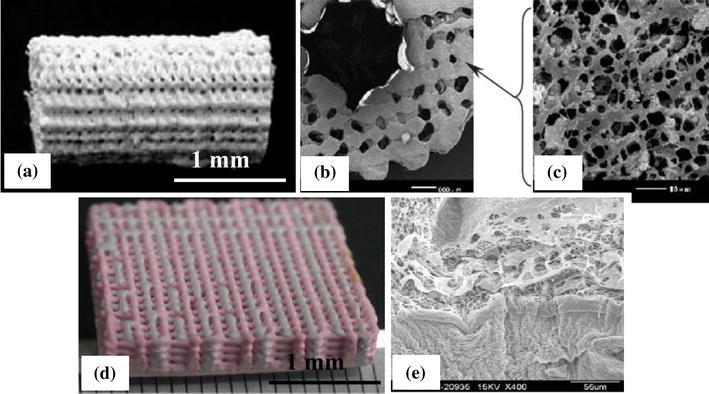
Images of **a** PLLA/TCP composite scaffold made in LDM process, SEM images of the cross-section of the scaffold; **b** low magnified; **c** high magnified (Xiong et al. 2002); **d** multi-material (PLGA/collagen) scaffold made in M-LDM process; and **e** SEM images of the interface of the scaffold (Liu et al. 2009)

To address the problem associated with the high-temperature effect on the polymeric biomaterial, another modified FDM process, LDM (Fig. 22) has been developed. LDM combines a nozzle extrusion process and a TIPS (Bartolo et al. 2008). The LDM developed by Xiong et al. (2002) involves the fabrication of 3D scaffolds in a low-temperature environment under 0 °C to solidify the material solution when deposited on the platform. Their PLLA/TCP composite scaffolds have a high porosity of up to 90 % as demonstrated in Fig. 23a–c. The mechanical strength values of the scaffolds were close to those of spongy human bone. The scaffolds were evaluated in vivo, showing good biocompatibility and good bone conductivity (Bartolo et al. 2008).

Independently, Li et al. (2011) developed an LDM system and fabricated PLGA/TCP scaffolds for alveolar bone repair. The composite scaffolds had porosity up to 87 % and the mechanical properties of the scaffolds were similar to cancellous bones. These scaffolds showed good biocompatibility in the attachment and proliferation of human bone marrow mesenchymal stem cells (HBMSC).

To fabricate scaffolds from heterogeneous materials, multi-nozzle low-temperature deposition and manufacturing (M-LDM) has been developed. This system offers great advantages in fabricating scaffolds with gradient porous structures and gradient biomolecules, which could potentially be used in the reconstruction of multi-tissue or complicated organs (Yan et al. 2003). Yan et al. (2003) fabricated bone tissue engineering scaffolds through single-nozzle deposition, bi-nozzle deposition and tri-nozzle deposition processes. M-LDM was recently applied by Liu et al. (2009) to fabricate composite scaffolds with two types of materials (i.e. PLGA, chitosan collagen and gelatine) and TCP via two nozzles, and a satisfactory combination of hydrophilic and mechanical properties was achieved (Fig. 23d–e).

With the elimination of the heat impact on biomaterials, LDM and M-LDM have the potential to fabricate the bioactive tissue scaffolds via the incorporation of biomolecules. The shortcoming of these two techniques is the necessity for solvent removal via a freeze-drying process, which is time consuming.

##### PED

**Fig. 24:**
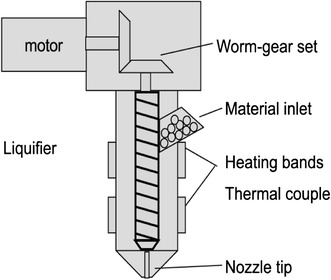
Schematic representation of PED (Wang et al. 2004)

To overcome the requirement of filament preparation in FDM, the modified FDM process known as PED (Fig. 24) was developed by Wang et al. (2004) for the fabrication of interconnected 3D scaffolds. This process employs raw material in the form of pellets that are fed into a chamber, and then directly extrudes scaffolding materials. Wang and co-workers directly fabricated cellular PCL scaffolds with a controlled pore size (~250 μm) and designed structural orientation without involving the material preparation and indirect casting. The results demonstrated that the strut width was consistent between samples, and all samples showed an interconnective porosity of higher than 98 %. The compression modulus of the scaffolds was in a range between 150 and 200 MPa.

**Fig. 25:**
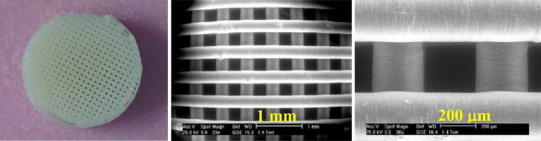
SEM images of **a** PCL scaffold fabricated via PED; **b** low magnified; and **c** high magnified (Shor et al. 2009)

Using PED, Shor et al. (2007) produced PCL and PCL/HA scaffolds that had a porosity of 60 and 70 % (respectively) and pore sizes of 450 and 750 μm (respectively). In vitro evaluation demonstrated that the fabrication process had no adverse cytotoxic effect on the scaffolds. Porous PCL scaffolds with a pore size of 350 μm and designed structural orientations (Fig. 25) were later produced by the same group (Shor et al. 2009). An in vivo study demonstrated that there was increasing osseous ingrowth during the 8-week culture period, indicating that the osteoblast cells were able to attach and proliferate on the scaffold (Shor et al. 2009). In addition, PED was used to produce composite scaffolds from poly(_L_-lactide-*co*-_D,L_-lactide) (PLDLLA)/TCP (Lam et al. 2009) and PCL/TCP (Arafat et al. 2011) for bone tissue engineering. The mechanical properties and in vitro cytocompatibility of the PED-fabricated composite scaffolds exhibited favourable results.

The PED process has several advantages over conventional FDM techniques in terms of no need of filament fabrication and the ability to print viscous materials. However, the major drawback of this technique is that it does not allow for incorporation of biomacromolecules and living cells into scaffolds during the processing, due to the elevated temperature that must be used to melt the materials. The heat impact at the elevated temperature may also damage polymeric materials during the processing.

##### PAM

**Fig. 26:**
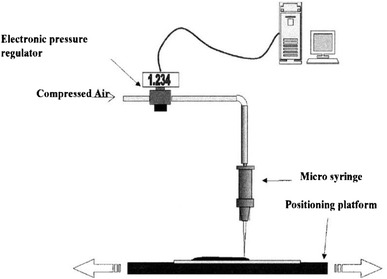
Schematic representation of PAM (Vozzi et al. 2002)

PAM (Fig. 26) is a modified FDM technique developed by Vozzi et al. (2002) that allows the fabrication of 3D scaffolds with a well-defined geometry at the micron scale. A solution of polymer can be extruded through a narrow capillary needle (diameter of between 10 and 20 μm) by the application of constant pressure of 20–300 mmHg. After the solvent is evaporated, the paste solidifies. By changing the syringe pressure, solution viscosity, diameter of the syringe tip and processing speed, the thickness of the materials deposited can be controlled. The higher the viscosity of the solutions used, the better are the resolutions that can be achieved. However, to extrude a solution of viscosities greater than approximately 400 cp demands high driving pressures, which may break the tip (Vozzi et al. 2003). This technique has been used for the deposition of a wide range of polymers and composites (Vozzi et al. 2003; Rattanakit et al. 2012; Tartarisco et al. 2009; Vozzi et al. 2013).

**Fig. 27:**
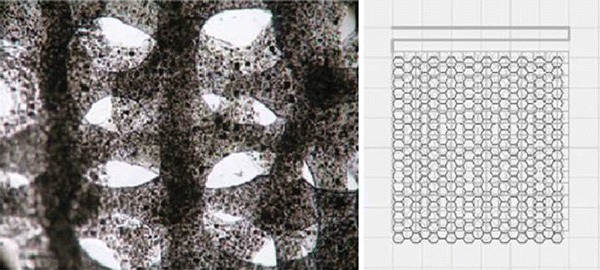
Light microscopy of the PAM-printed PLLA/CNT composite scaffolds (Vozzi et al. 2013)

PAM was used to produce PLGA scaffolds with high lateral resolution (10–30 μm feature) (Vozzi et al. 2003). 3D PLLA/carbon nanotubes (CNTs) composite scaffolds (Fig. 27) have been developed by Vozzi et al. (2013) for bone tissue engineering. The composite structures exhibited improvement in the mechanical properties in comparison with the pure 3D PLLA scaffolds. In vitro cell culture of the scaffolds also showed that they support osteoblast proliferation.

More recently, the same group further developed PAM with a new model, piston-assisted microsyringe known as ‘PAM2’ (Vozzi and Ahluwalia 2007; Tirella et al. 2011; Vozzi et al. 2012), which aimed at the microfabrication of cell-incorporated hydrogels. Instead of using the air pressure, the new process uses a mechanical piston as the driving force for extrusion, which allows the control of the material outflow from the needle tip. Low shear stresses over short periods are involved during the ejection of cells without considerable damage of cell membrane. In short, PAM2 can print the well-defined structures of highly viscous materials incorporated with cells.

The major advantages of this technique are its simplicity and its fabrication of well-defined 3D scaffolds in a variety of patterns and with a wide range of thicknesses. It can produce structures with the highest lateral resolution of 5–10 μm. The operating system at low temperature also allows for the incorporation of proteins and other biomolecules, which can build favourable microenvironments for tissue regeneration. The limitations of this technique are the low vertical dimension, the inability to incorporate even small particles, and the limited usage for low-concentrated solutions. The last two drawbacks are due to clogging of the syringe needle.

##### Robocasting

**Fig. 28:**
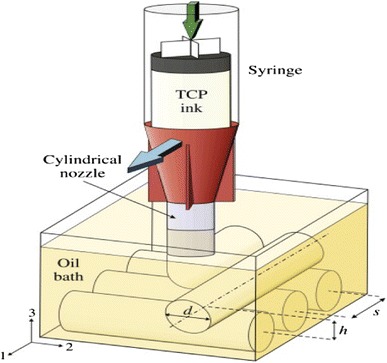
Schematic representation of robocasting (Martínez-Vázquez et al. 2010)

Robocasting, also referred to as ‘robotic deposition’ and ‘direct-write assembly’ (Fig. 28), was developed at the Sandia National Laboratory (Cesarano et al. 1998). This technique can lay down a highly concentrated, pseudoplastic-like colloidal suspension (water-based inks) through a small nozzle inside a non-wetting oil bath. The soft pseudoplastic then becomes a rigid mass after the evaporation of water from the paste (Smay et al. 2002). This technique has been used to produce porous ceramic and composite scaffolds with different architectures (Miranda et al. 2006; Hoelzle et al. 2008; Miranda et al. 2008; Martínez-Vázquez et al. 2010).

**Fig. 29:**
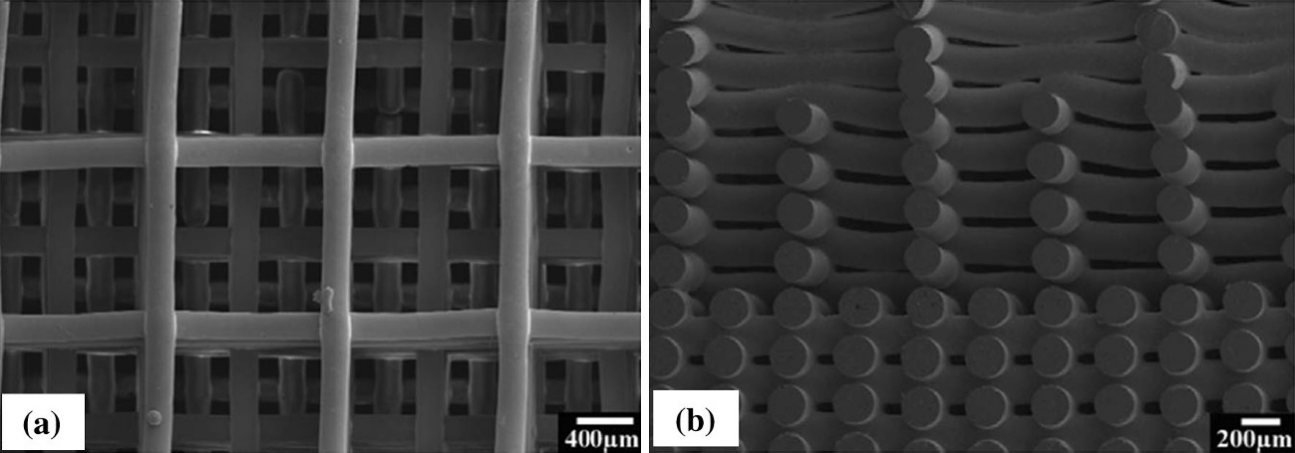
SEM images of **a** surface view of a glass (6P53B) scaffold with a gradient pore size; and **b** cross sections of the scaffold (Fu et al. 2011)

Using the robocasting technique, Fu et al. (2011) prepared bioglass scaffolds from the suspension of bioactive glass (6P53B) in Pluronic F-127 aqueous solution. The sintered glass scaffolds with 60 % porosity showed a compressive strength (136 ± 22 MPa) comparable to that of human cortical bone (100-150 MPa), which is suitable for load-bearing applications (Fig. 29). In addition, Dellinger et al. (2006) produced model HA scaffolds of various architectures, including periodic, radial, and super-lattice structures with macropores (100–600 μm), micropores (1–30 μm), and submicron pores (<1 μm). This study indicates that by precise control of scaffold features, these model scaffolds may be used to systematically study the effects of scaffold porosity on bone ingrowth processes both in vitro and in vivo.

Heo et al. (2009) produced HA/PCL composite scaffolds using the robocasting process. The macropores in the scaffolds were well interconnected, with a porosity of 73 % and a pore size of 500 μm. The compressive modulus of the nano-HA/PCL and micro-HA/PCL scaffolds was 3.2 and 1.3 MPa, respectively. The higher modulus of nano-HA/PCL was to be likely caused by the well-dispersed nanosized HA particles. In addition, the more hydrophilic surface of nano-HA/PCL, which resulted from the greater surface area of HA of nano size, could promote cell attachment and proliferation compared with micro-HA/PCL. Martinez-Vazquez et al. (2010) reported the infiltration of PCL or PLA into β-TCP porous scaffolds fabricated by robocasting increased their compressive strength compared with pure calcium phosphate scaffolds.

(PLA or PCL)/(HA, CaP or bioactive glass) composite scaffolds were fabricated in the robocasting process with inorganic contents as large as 70 wt % (Russias et al. 2007; Serra et al. 2013). The addition of PEG to PLA matrix, combined with other processing parameters, could reduce the ink viscosity and thus allow for printing high-resolution 3D scaffolds. All these scaffolds showed encouraging biological response in in vitro evaluations.

Robocasting is a versatile technique that allows the printing of a range of materials and the fabrication of scaffolds with a range of architectures spanning distances up to 1 mm. With the ability of fully supporting its own weight of suspensions during assembly, the scaffold fabrication requires no sacrificial support material or mould (Smay et al. 2002). However, the optimisation of ceramic inks suitable for direct print assembly is a primary concern. This is because if a ceramic ink contains a content of ceramic powders that is too low, it will dry quickly resulting in microcracks in the products (Miranda et al. 2006).

##### 3D-Bioplotter^®^

**Fig. 30:**
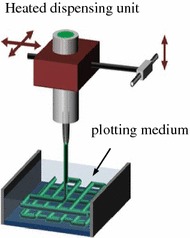
Schematic representation of 3D-Bioplotter^®^ (Landers et al. 2002; Gurr and Mülhaupt 2012)

3D-Bioplotter^®^ (Fig. 30) is another variant of the FDM technique for fabricating scaffolds, especially for the soft-tissue engineering purposes. This technique was developed by the researchers at the Freiburg Materials Research Centre (Landers et al. 2002; Gurr and Mülhaupt 2012). While other extrusion-based methods deposit materials onto a solid platform, this technique involves moving an extruder head to dispense plotting material into a liquid medium. The extruder head is made of a micro needle and a cartridge where liquids, solutions, dispersion polymers, pastes, hot melts or reactive oligomers are initially stored. The plotting material solidifies in the liquid medium, which compensates the gravity with a buoyancy force. As a result, no support structure is required.

**Fig. 31:**
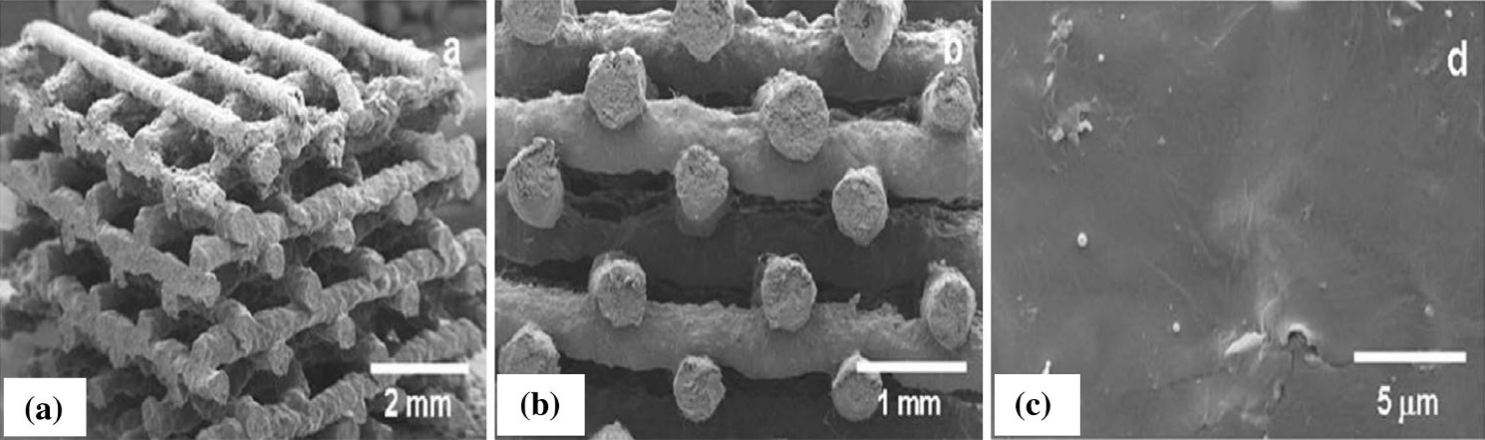
SEM micrographs of **a** a scaffold obtained with the 3D-Bioplotter^®^ technology; **b** cross-sectional view of the scaffold; and **c** the surface morphology (Oliveira et al. 2010)

Using the 3D-Bioplotter^®^ technique, Lander and Pfister (Landers et al. 2002) printed alginate/fibrin hydrogel scaffolds with internal pore sizes of 200–400 μm and porosity of 40–50 %. The hydrogel scaffolds were further surface coated to facilitate cell adhesion and cell growth (Landers et al. 2002). Lode et al. (2012) have recently produced HA cement scaffolds using the 3D-Bioplotter^®^ technique. To date, this technique has been used to fabricate scaffolds from a number of biomaterials, including PCL (Oliveira et al. 2009; Ye et al. 2010), PLGA (Daoud et al. 2011), poly(ethylene oxide terephthalate)-*co*-poly(butylene terephthalate) (PEOT/PBT) (Bettahalli et al. 2013), and starch-based blends (Martins et al. 2009; Sobral et al. 2011; Oliveira et al. 2010). Figure 31 presents the example of starch-based scaffolds fabricated via the 3D-Bioplotter^®^ system.

The usage of the 3D-Bioplotter^®^system offers opportunities for fabricating scaffolds from a broad range of materials and for incorporating biological entities such as biomolecules, proteins, and even living cells, into the structures under the physiologically relevant temperature. The major drawbacks of this technique are that the hydrogels produced by 3D-Bioplotter^®^ have a limited resolution, lack mechanical strength, and have a smooth surface that might be non-adherent for cells (Landers et al. 2002).

## Comparison of scaffolding techniques

**Fig. 32:**
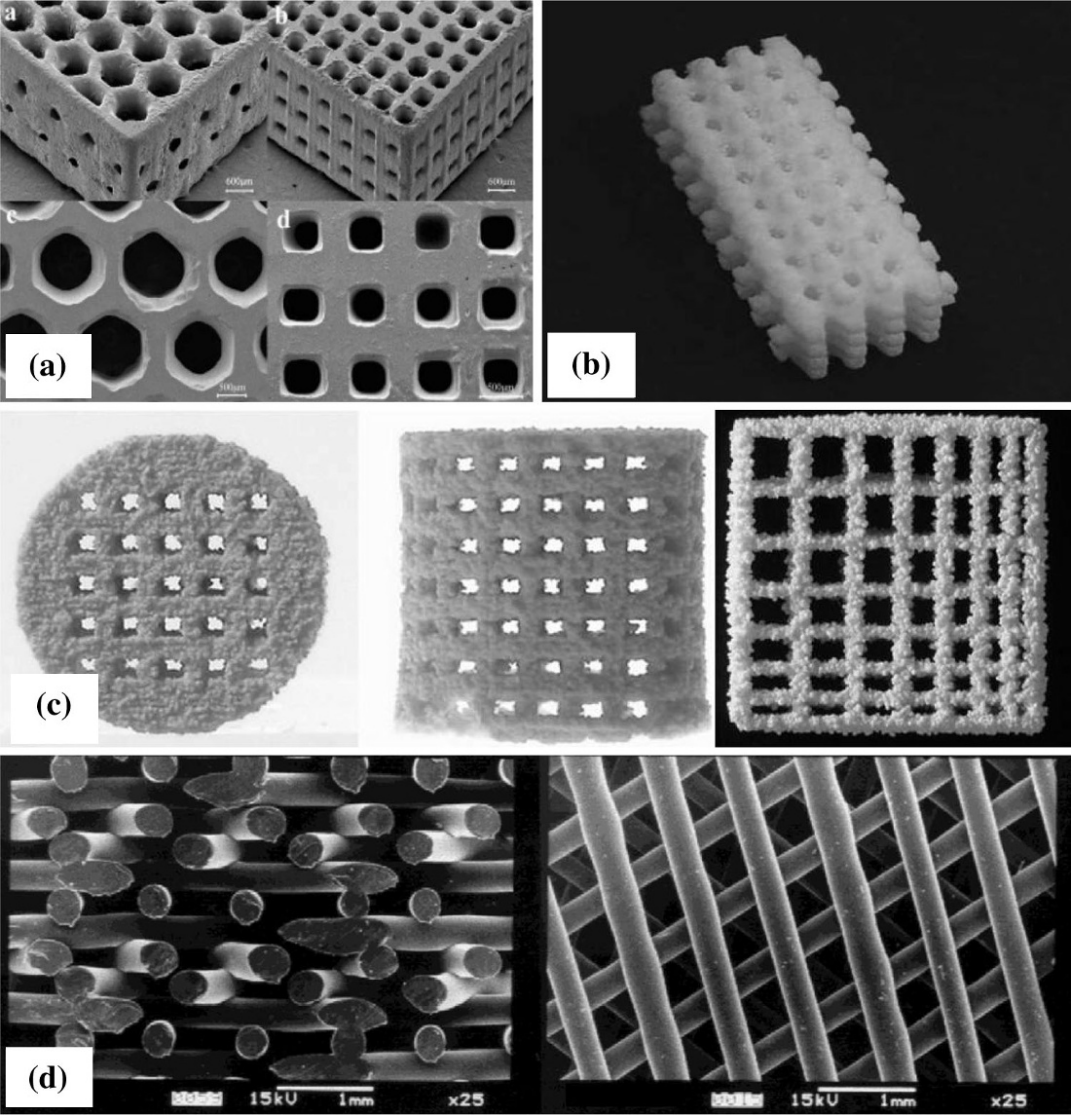
3D scaffolds manufactured by various SFF techniques: **a** SLA; **b** SLS; **c** 3DP; and **d** FDM (Dalton et al. 2009)

Among various SFF techniques, SLA, SLS, 3DP and FDM have been used in the scaffold fabrication, especially for applications in bone tissue engineering. Figure 32 shows typical structures of porous scaffolds produced by different SFF techniques. This section aims to provide a comparison of the above four systems.

### SLA

SLA offers several advantages over other SFF techniques for scaffold fabrication:

1. SLA offers high spatial accuracy at dimensional resolutions below 50 μm.
2. The feature of size can be possible at below 1 μm.
3. SLA provides a high-surface quality of parts.

However, there are several limitations of SLA, including:

1. SLA requires expensive machinery.
2. SLA requires support structures to prevent damage to the part surface when removed.
3. The construction time involved in SLA could be lengthy, depending on the design resolution and size.
4. The choice of photo-sensitive resins available in commercial markets is limited, and most resins are toxic to cells.

### SLS

SLS offers several benefits over other SFF techniques for scaffold fabrication:

1. The SLS process allows for the fabrication of scaffolds with a controlled structure.
2. A wide range of biomaterials (polymers, ceramics and composites) can be processed.
3. SLS can fabricate highly interconnected porous scaffolds with a pore size of 50 μm or less.
4. SLS does not need support structures or organic solvents.

The limitations of SLS include the following:

1. The high temperature in the bed powder can allow the thermal degradation of materials.
2. The resolution of the SLS system is limited by the shape, size and size distribution of powders used.
3. Removing unprocessed powders trapped into the small hole of scaffolds is difficult.

### 3D-printing

3DP offers several benefits over other SFF techniques for scaffold fabrication:

1. 3DP can create scaffolds with high consistency and controlled structural anisotropy.
2. 3DP does not involve high temperature, harsh chemicals, and support structures.
3. The high building speed of the print head makes the mass production of scaffolds possible.
4. It is possible to incorporate biological agents into the scaffolds if the binder is water.

The limitations of the 3DP process include the following:

1. The layer thickness relies on the particle size of the powder used.
2. 3DP-fabricated scaffolds have a rough and ribbed surface finish, which affects the resolution and accuracy of the parts.
3. The scaffolds are relatively fragile and lack mechanical stability.
4. The unprocessed powders trapped in small pores of the parts are difficult to remove.

### FDM

FDM offers several benefits over other SFF techniques for scaffold fabrication:

1. A high degree of precision can be achieved in the *x*–*y* direction.
2. The versatility in the number of lay-down patterns allows for freedom to print materials in any direction within each consecutive layer of an FDM structure.
3. The FDM technique is capable of fabricating scaffolds with good structural integrity and mechanical stability because of the proper fusion between individual material layers.
4. Material wastage is minimal because of direct extrusion.

The limitations of the FDM process include:

1. FDM is limited to the use of filament materials with good melt viscosity properties.
2. There is a need to use a high temperature to melt materials, which results in damage to materials.
3. Control of the *z* direction can be difficult.
4. The support structure required during processing is rather difficult to remove, and may cause the risk of material contamination.
5. Mass production of scaffolds is difficult due to its slow build speed.

Table 10 summarises the resolution, accuracy, porous structures and mechanical property of the 3D scaffolds produced through different SFF techniques, as well as the advantages and disadvantages of each SFF technique.

**Table 10:** Summary of key characteristics, advantages and disadvantages of SFF scaffolding techniques

Technique	Layer thickness ($\varvec{\upmu}$m)	Resolution ($\varvec{\upmu}$m)	Typical accuracy ($\varvec{\upmu}$m)		Porosity (%) and pore size ($\varvec{\upmu}$m)		Advantages	Disadvantages		Ref.
Photopolymerisation-based processing										
Stereolithography (SLA)	25–150	14–150	<50		<90 20–1,000		Good surface finish; possibly build transparent parts; excellent accuracy; anatomically shaped structures	Expensive machinery; support structure needed; the limited choice of resin available; use of mostly toxic resins; shrinkage during polymerisation		(Melchels et al. 2012; Dalton et al. 2009; Gurr and Mülhaupt 2012; Mota et al. 2012; Swift and Booker 2013)
Micro-stereolithography (μSLA)	<1	0.5–10	0.2		– 100–300		Similar to SLS; the highest resolution with micrometre scale	Similar to SLA		(Choi et al. 2009; Seol et al. 2013; Mota et al. 2012)
Two-photon polymerisation (TPP)	<5	0.1–4	0.2		–		Similar to SLS; low laser intensity; very fine lateral resolutions; fast processing	Similar to SLA		(Melchels et al. 2010; Weiss et al. 2009; Weiss et al. 2011; Mota et al. 2012)
Digital light processing (DLP)	15-70	40	<0.4		<90 500		Similar to SLS; no use of laser; higher resolution; higher build speed	Similar to SLA		(Felzmann et al. 2012; Tesavibul et al. 2012)
Powder-based processing										
Selective laser sintering (SLS)	75-150	50-1000	50-100		<40 30–2,500		Solvent free; no need for support material; fast processing	Expensive machinery; difficulty removing trapped powder; high temperatures in the chamber; powdery surface finish		(Melchels et al. 2012; Leong et al. 2003; Dalton et al. 2009; Gurr and Mülhaupt 2012; Mota et al. 2012; Swift and Booker 2013)
Surface selective laser sintering (SSLS)	200	150-200	<20		–		Similar to SLS; reduction of heat operating temperature; possible incorporation of bioactive agents	Similar to SLS		(Antonov et al. 2005; Kanczler et al. 2009)
Three-dimensional printing (3DP)	50-150	50-300	50-100		<45–60 45–1,600		Easy process; low cost; low heat effect on raw powder; no need for support material; fast processing	Poor surface finish, accuracy and mechanical properties; difficulty removing trapped powder; powdery surface finish		(Melchels et al. 2012; Leong et al. 2003; Dalton et al. 2009; Gurr and Mülhaupt 2012; Mota et al. 2012; Swift and Booker 2013)
Extrusion-based processing										
Fused deposition modelling (FDM)	50–750	100–500		100	<80 100–2,000	Solvent free; no materials trapped in the scaffolds; good mechanical strength; wide range of materials; versatile in lay-down pattern; low costs			Needs filament preparation; limited choice of filament materials; high heat effect on material; difficult fabrication for scaffolds with small pore sizes; medium accuracy	(Melchels et al. 2012; Leong et al. 2003; Dalton et al. 2009; Mota et al. 2012; Swift and Booker 2013)
Multi-head deposition system (MHDS)	200	several tens of microns		several tens of microns	~70 600	Enhanced range of material use and pore architecture; high resolution			High heat effect on material	(Kim and Cho 2009)
Low-temperature deposition manufacturing (LDM) and (M-LDM)	150	300-500			~88 200–500	Enhanced range of material use; ability to incorporate biomolecules			Solvent use; requires freeze drying	(Yeong et al. 2004; Xiong et al. 2002; Li et al. 2011; Liu et al. 2009; Mota et al. 2012)
Precision extruding deposition (PED)	250	100–500		100	<70 200–500	No requirement of filament preparation			High heat effect on material; rigid filament	(Melchels et al. 2012; Yeong et al. 2004; Shor et al. 2009; Arafat et al. 2011; Mota et al. 2012)
Pressure-assisted microsyringe (PAM)/(PAM2)	150–200	10–1000		5–10	70 10-600	Enhanced range of material use; ability to incorporate biomolecules; very fine resolution			Small nozzle inhibits incorporation of particles; narrow range of printable viscosities; solvent use	(Yeong et al. 2004; Tartarisco et al. 2009; Vozzi and Ahluwalia 2007; Heo et al. 2009; Mota et al. 2012)
Robocasting (direct-write assembly)	250	100–450		few microns	<90 5–100	Enhanced range of material use; possible fabrication of highly concentrated suspension; no need of support material; excellent resolution			Expensive machinery; precise control of ink properties is crucial	(Melchels et al. 2012; Yeong et al. 2004; Serra et al. 2013; Mota et al. 2012)
3D-Bioplotter^®^	50–300	100–500		100	– 200–400	Enhanced range of material use and conditions; ability to incorporate biomolecules, proteins and cells			Low strength; smooth surface; low accuracy; slow processing; calibration for new material; suitability for soft-tissue area	(Yeong et al. 2004; Landers et al. 2002; Gurr and Mülhaupt 2012; Mota et al. 2012)

## Summary

The use of conventional fabrication techniques (such as solvent casting in combination with particulate leaching, gas foaming, phase separation, freeze-drying, electrospinning, powder-forming processes and sol–gel techniques) for ceramic fabrication has a limited capacity to control the internal and external architecture of scaffolds in dimension, pore morphology, pore size, pore interconnectivity and overall porosity. The scaffolds fabricated using the conventional methods suffer from insufficient mechanical integrity.

SFF offers several benefits over conventional fabrication techniques, including high flexibility in shape and size, capabilities of precise control over spatial distribution, high reproducibility, and suitability to a broad variety of biomaterials, and customised design with specific patient needs. Currently, SLA, SLS, 3DP and FDM are most frequently used in the fabrication of scaffolds from polymers, ceramics and their composites. Depending on the type of materials and their specific form, each SFF technique provides unique internal and external features of scaffold architectures. Among these SFF techniques, SLA and FDM have unique benefits and have been extensively studied for producing 3D structures that enable good mechanical and biological properties throughout the entire scaffold. SLA can create tissue engineering scaffolds with excellent accuracy and good surface quality. FDM offers versatile fabrication in lay-down pattern and good structural integrity. In addition, both systems are able to fabricate parts with good mechanical integrity (Table 11**)** over the powder-based system (3DP) due to proper fusion bonding between individual material layers.

**Table 11:** Comparison of mechanical properties in different RP techniques

Technique	Scaffolding materials	Tensile strength (MPa)	Compressive strength (MPa)	References
Stereolithography (SLA)	Liquid form of photopolymer	Up to 75	~57	(Swift and Booker 2013; Kim and Oh 2008)
Selective laser sintering (SLS)	Thermoplastics	~50	60–75	(Swift and Booker 2013; Kim and Oh 2008)
Three-dimensional printing (3DP)	Plastic powder	<5	<5	(Swift and Booker 2013; Kim and Oh 2008)
Fused deposition modelling (FDM)	Thermoplastics	35–60	45–70	(Swift and Booker 2013; Kim and Oh 2008)

## References

[CR1] Chen A, Tsang V, Albrecht D, Bhatia S (2007) 3-D fabrication technology for tissue engineering. In: Ferrari M, Desai T, Bhatia S (eds) BioMEMS and biomedical nanotechnology. Springer, Berlin, pp 23–38

[CR2] Oliveira AL, Costa SA, Sousa RA, Reis RL (2009) Nucleation and growth of biomimetic apatite layers on 3D plotted biodegradable polymeric scaffolds: effect of static and dynamic coating conditions. Acta Biomaterialia 5:1626–1638 10.1016/j.actbio.2008.12.009 19188103

[CR3] Lode A, Meissner K, Luo Y, Sonntag F, Glorius S, Nies B, Vater C, Despang F, Hanke T, Gelinsky M (2012) Fabrication of porous scaffolds by three-dimensional plotting of a pasty calcium phosphate bone cement under mild conditions. J Tissue Eng Regenerat Med 10.1002/term.1563 22933381

[CR4] Martins A, Chung S, Pedro AJ, Sousa RA, Marques AP, Reis RL, Neves NM (2009). Hierarchical starch-based fibrous scaffold for bone tissue engineering applications. J Tissue Eng Regenerat Med.

[CR5] Duan B, Wang M (2010). Customized CaP/PHBV nanocomposite scaffolds for bone tissue engineering: design, fabrication, surface modification and sustained release of growth factor. J R Soc Interf.

[CR6] Duan B, Wang M, Zhou WY, Cheung WL, Li ZY, Lu WW (2010). Three-dimensional nanocomposite scaffolds fabricated via selective laser sintering for bone tissue engineering. Acta Biomaterialia.

[CR7] Sundback CA, Shyu JY, Wang Y, Faquin WC, Langer RS, Vacanti JP, Hadlock TA (2010). Biocompatibility analysis of poly(glycerol sebacate) as a nerve guide material. Biomaterials.

[CR8] Vacanti CA (2000) Foreword. In: Lanza RP, Langer R, Vacanti JP (eds) Principles of tissue engineering, 2 edn. Academic Press, California, p xxix

[CR9] Vacanti CA, Bonassar LJ, Vacanti JP (2000) Structure tissue engineering. In: Lanza RP, Langer R, Vacanti JP (eds) Principles of tissue engineering, 2 edn. Academic Press, California, pp 671–682

[CR10] Brown CD, Hoffman AS (2002) Modification of natural polymer: Chitosan. In: Atala A, Lanza RP (eds) Methods of tissue engineering. Academic Press, California, pp 565–574

[CR11] Mota C, Puppi D, Chiellini F, Chiellini E (2012) Additive manufacturing techniques for the production of tissue engineering constructs. J Tissue Eng Regenerat Med 10.1002/term.1635 23172792

[CR12] Lam CXF, Olkowski R, Swieszkowski W, Tan KC, Gibson I, Hutmacher DW (2009) Composite PLDLLA/TCP scaffolds for bone engineering: mechanical and in vitro evaluations. In: The 13th international conference on biomedical engineering, Singapore, pp 1480–1483

[CR13] Mooney DJ, Baldwin DF, Suh NP, Vacanti JP, Langer R (1996). Novel approach to fabricate porous sponges of poly(d,l-lactic-co-glycolic acid) without the use of organic solvents. Biomaterials.

[CR14] Stuckey DJ, Ishii H, Chen QZ, Boccaccini AR, Hansen U, Carr CA, Roether JA, Jawad H, Tyler DJ, Ali NN, Clarke K, Harding SE (2010). Magnetic resonance imaging evaluation of remodeling by cardiac elastomeric tissue scaffold biomaterials in a rat model of myocardial infarction. Tissue Eng Part A Tissue Eng.

[CR15] Hutmacher DW, Hoque ME, Wong YS (2008) Design, fabrication and physical characterization of scaffolds made from biodegradable synthetic polymers in combination with RP systems based on melt extrusion. In: Bidanda B, Bártolo PJ (eds) Virtual prototyping & bio manufacturing in medical applications. Springer, New York, pp 261–291

[CR16] Melchels FPW, Barradas AMC, Blitterswijk CAV, Boer JD, Feijen J, Grijpma DW (2010). Effects of the architecture of tissue engineering scaffolds on cell seeding and culturing. Acta Biomaterialia.

[CR17] Kim GD, Oh YT (2008). A benchmark study on rapid prototyping processes and machines: quantitative comparisons of mechanical properties, accuracy, roughness, speed, and material cost. Proc Inst Mech Eng Part B J Eng Manuf.

[CR18] Lee G, Barlow JW (1993) Selective laser sintering of bioceramic materials for implants. In: Proceedings of solid freeform fabrication symposium, Austin, TX, pp 376–380

[CR19] Tartarisco G, Gallone G, Carpi F, Vozzi G (2009). Polyurethane unimorph bender microfabricated with Pressure Assisted Microsyringe (PAM) for biomedical applications. Mater Sci Eng C.

[CR20] Vozzi G, Tirella A, Ahluwalia A (2012) Rapid prototyping composite and complex scaffolds with PAM2. Methods Mol Biol (Clifton, N.J.) 868:57–69 10.1007/978-1-61779-764-4_4 22692604

[CR21] Cornejo IA, McNulty TF, Lee SY, Bianchi E, Danforth SC, Safari A (2000) Development of bioceramic tissue scaffolds via fused deposition of ceramics. In: George L (ed) Bioceramics: materials and applications III. American Ceramic Society, Westerville, pp 183–195

[CR22] Reichert JC, Hutmacher DW (2011) Bone tissue engineering. In: *Tissue Engineering*, N. Pallua and C. V. Suschek, Eds., ed New York: Springer, 2011, pp. 431-456

[CR23] Cesarano J, Segalman R, Calvert P (1998). Robocasting provides moldless fabrication from slurry deposition. Ceram Ind.

[CR24] Smay JE, Lewis JA (2012) Solid free-form fabrication of 3-D ceramic structures. In: Bansal NP, Boccaccini AR (eds) Ceramics and composites processing methods, 1st edn. Wiley, New Jersey, pp 459–484

[CR25] Jansen J, Melchels FPW, Grijpma DW, Feijen J (2008) Fumaric acid monoethyl ester-functionalized poly(_D,L_-lactide)/*N*-vinyl-2-pyrrolidone resins for the preparation of tissue engineering scaffolds by stereolithography. Biomacromolecules 10:214–220 10.1021/bm801001r 19090782

[CR26] Stuecker JN, Cesarano Iii J, Hirschfeld DA (2003). Control of the viscous behavior of highly concentrated mullite suspensions for robocasting. J Mater Process Technol.

[CR27] Temenoff JS, Lu L, Mikos AG (2000) Bone tissue engineering using synthetic biodegradable polymer scaffolds. In: Davies JE (ed) Bone engineering. EM Squared, Toronto, pp 455–462

[CR28] Swift KG, Booker JD (2013) Rapid prototyping processes. In: Manufacturing process selection handbook. Butterworth-Heinemann, Oxford, pp 227–241

[CR29] Schwartzalder K, Somers AV (1963) Method of making a porous shape of sintered refractory ceramic articles

[CR30] Hench LL (1999) Bioactive glasses and glasses–ceramics. In: Shackelford JF (ed) Biocaramics-applications of ceramic and glass materials in medicine. Trans Tech Publication, Switzerland, pp 37–64

[CR31] Ye L, Zeng X, Li H, Ai Y (2010) Fabrication and biocompatibility of nano non-stoichiometric apatite and poly(ε-caprolactone) composite scaffold by using prototyping controlled process. J Mater Sci Mater Med 21:753–760 10.1007/s10856-009-3872-4 19784867

[CR32] Murphy MB, Mikos AG (2007) Polymer scaffold fabrication. In: Lanza R, Langer R, Vacanti J (eds) Principles of tissue engineering, 3 edn. Academic Press, California, pp 309–321

[CR33] O’Donnell MD (2012) Melt-derived bioactive glass. In: Jones JR, Clare AG (eds) Bio-glasses: an introduction. Wiley, West Sussex

[CR34] Gurr M, Mülhaupt R (2012) Rapid prototyping. In: Krzysztof M, Martin M (eds) Polymer science: a comprehensive reference. Elsevier, Amsterdam, pp 77–99

[CR35] Potoczek M, Zima A, Paszkiewicz Z, Slosarczyk A (2009). Manufacturing of highly porous calcium phosphate bioceramics via gel-casting using agarose. Ceram Int.

[CR36] Hopkinson N, Dickens P (2006) Emerging rapid manufacturing processes. In: Hopkinson N, Hague RJM, Dickens PM (eds) Rapid manufacturing: an industrial revolution for the digital age. Wiley, West Sussex, pp 55–80

[CR37] Dalton PD, Woodfield T, Hutmacher DW (2009). Snapshot: polymer scaffolds for tissue engineering. Biomaterials.

[CR38] Bartolo PJ, Almeida HA, Rezende RA, Laoui T, Bidanda B (2008) Advanced processes to fabricate scaffolds for tissue engineering. In: Bidanda B, Bartolo PJ (eds) Virtual prototyping of biomanufacturing in medical application. Springer, New York, pp 149–170

[CR39] Wolfe PS, Sell SA, Bowlin GL (2011) Natural and synthetic scaffolds. In: Pallua N, Suschek CV (eds) Tissue engineering. Springer, New York, pp 41–67

[CR40] Ma PX, Langer R (1995) Degradation, structure and properties of fibrous poly(glycolic acid) scaffolds for tissue engineering. In: Mikos AG (ed) Polymers in medicine and pharmacy, vol 394. Materials Research Society, Pennsylvania, pp 99–110

[CR41] Chen Q, Liang S, Thouas GA (2013) Elastomeric biomaterials for tissue engineering. Progress in polymer science, vol 38, pp 584–671

[CR42] Fu Q, Saiz E, Tomsia AP (2011). Direct ink writing of highly porous and strong glass scaffolds for load-bearing bone defects repair and regeneration. Acta Biomaterialia.

[CR43] Chen QZ (2007) Bioglass^®^-derived glass-ceramic scaffolds for bone tissue engineering. PhD, Materials, Imperial College London, London

[CR44] Chen QZ, Roether JA, Boccaccini AR (2008) Tissue engineering scaffolds from bioactive glass and composite materials. In: Ashammakhi N, Reis R, Chiellini F (eds) Topics in tissue engineering, vol 4. BTE group, pp 1–23

[CR45] Chen Q-Z, Bismarck A, Hansen U, Junaid S, Tran MQ, Harding SE, Ali NN, Boccaccini AR (2008). Characterisation of a soft elastomer poly(glycerol sebacate) designed to match the mechanical properties of myocardial tissue. Biomaterials.

[CR46] Chen Q-Z, Harding SE, Ali NN, Lyon AR, Boccaccini AR (2008) Biomaterials in cardiac tissue engineering: ten years of research survey. Mater Sci Eng R Rep 59:1–37

[CR47] Muzzarelli RAA, Muzzarelli C (2005) Chitosan chemistry: relevance to the biomedical sciences. In: Polysaccharides 1: structure, characterization and use. Springer, Berlin, pp 151–209

[CR48] Kohn J, Langer R (1996) Bioresorbable and bioerodible materials. In: Ratner BD, Hoffman AS, Schoen FJ, Lemons JE (eds) Biomaterials science: an introduction to materials in medicine. Academic Press, New York, pp 64–72

[CR49] Danforth S, Safari A, JMA, Langrana N (1998) Solid free form fabrication (SFF) of functional advanced ceramic components. Naval research review, office of naval research three, vol L, pp 27–38

[CR50] Hollister SJ (2005). Porous scaffold design for tissue engineering. Nat Mater.

[CR51] Onagoruwa S, Bose S, Bandyopadhyay A (2001) Fused deposition of ceramics (FDC) and composites. In: Solid freeform fabrication symposium, The University of Texas at Austin, pp 224–231

[CR52] Padilla S, Sánchez-Salcedo S, Vallet-Regí M (2006) Bioactive glass as precursor of designed-architecture scaffolds for tissue engineering. J Biomed Mater Res 81A:224–232 10.1002/jbm.a.30934 17120207

[CR53] Liang S-L, Cook WD, Thouas GA, Chen Q-Z (2010) The mechanical characteristics and in vitro biocompatibility of poly(glycerol sebacate)-Bioglass^®^ elastomeric composites. Biomaterials 31:8516–8529 10.1016/j.biomaterials.2010.07.105 20739061

[CR54] Pereira TF, Oliveira MF, Maia IA, Silva JVL, Costa MF, Thiré RMSM (2012) 3D printing of poly(3-hydroxybutyrate) porous structures using selective laser sintering. Macromolecular Symposia 319:64–73

[CR55] Grimm T (2004) User’s guide to rapid prototyping. Society of Manufacturing Engineers (SME), Dearborn, pp 367–396

[CR56] Chu TM, Halloran JW, Wagner WC (1997) Hydroxyapatite suspension for implant fabrication by stereolithography. In: Ghosh A, Barks RE, Hiremath B (eds) Case studies in ceramic product development. American Ceramic Society, Westerville, pp 119–125

[CR57] Chu TM, Halloran JW, Wagner WC (1996) Ultraviolet curing of highly loaded hydroxyapatite suspension. In: Rusin RP, Fischman GS (eds) Bioceramics: materials and applications II. American Ceramic Society, Westerville, pp 57–66

[CR58] Niemelä T, Kellomäki M (2011) Bioactive glass and biodegradable polymer composites. In: Ylänen HO (ed) Bioactive glasses: materials, properties and applications. Woodhead Publishing Limited, Cambridge, pp 227–245

[CR59] Chu TMG (2006) Solid freeform fabrication of tissue engineering scaffolds. In: Ma PX, Elisseeff J (eds) Scaffolding in tissue engineering. CRC Press, Florida, pp 139–153

[CR60] Popov VK, Antonov EN, Bagratashvili VN, Konovalov AN, Howdle SM (2004) Selective laser sintering of 3-D biodegradable scaffolds for tissue engineering. In: Materials research society symposium proceeding, pp F.5.4.1–F.5.4.3

[CR61] Correlo VM, Oliveira JM, Mano JF, Neves NM, Reis RL (2011) Natural origin materials for bone tissue engineering e properties, processing, and performance. In: Atala A, Lanza R, Thomson JA, Nerem RM (eds) Principles of regenerative medicine, 2 edn. Academic Press, Canada, pp 557–586

[CR62] Sun W, Starly B, Nam J, Darling A (2005) Bio-CAD modeling and its applications in computer-aided tissue engineering. Comput-Aided Des 37:1097–1114

[CR63] Morsi YS, Wong CS, Patel SS (2008) Conventional manufacturing processes for three-dimensional scaffolds. In: Bidanda B, Bartolo PJ (eds) Virtual prototyping of biomanufacturing in medical application. Springer, New York, pp 129–148

[CR64] Andrade JCT, Camilli JA, Kawachi EY, Bertran CA (2002). Behavior of dense and porous hydroxyapatite implants and tissue response in rat femoral defects. J Biomed Mater Res.

[CR65] Anithaa R, Arunachalam S, Radhakrishnan P (2001). Critical parameters in ¯uencing the quality of prototypes in fused deposition modelling. J Mater Process Technol.

[CR66] Antonov EN, Bagratashvili VN, Whitaker MJ, Barry JJA, Shakesheff KM, Konovalov AN, Popov VK, Howdle SM (2005). Three-dimensional bioactive and biodegradable scaffolds fabricated by surface-selective laser sintering. Adv Mater.

[CR67] Arafat MT, Lam CXF, Ekaputra AK, Wong SY, Li X, Gibson I (2011). Biomimetic composite coating on rapid prototyped scaffolds for bone tissue engineering. Acta Biomater.

[CR68] Arcaute K, Mann B, Wicker R (2010). Stereolithography of spatially controlled multi-material bioactive poly(ethylene glycol) scaffolds. Acta Biomater.

[CR69] Attawin MA, Herbert KM, Laurencin CT (1995). Osteoblast-like cell adherence and migration through 3-dimensional porous polymer matrices. Biochem Biophys Res Commun.

[CR70] Bael SV, Desmet T, Chai YC, Pyka G, Dubruel P, Kruth J-P, Schrooten J (2013). *In vitro* cell-biological performance and structural characterization of selective laser sintered and plasma surface functionalized polycaprolactone scaffolds for bone regeneration. Mater Sci Eng C.

[CR71] Baino F, Vitale-Brovarone C (2011). Three-dimensional glass-derived scaffolds for bone tissue engineering: current trends and forecasts for the future. J Biomed Mater Res Part A.

[CR72] Barroca N, Daniel-da-Silva AL, Vilarinho PM, Fernandes MHV (2010). Tailoring the morphology of high molecular weight PLLA scaffolds through bioglass addition. Acta Biomater.

[CR73] Bergmann C, Lindner M, Zhang W, Koczur K, Kirsten A, Telle R, Fischer H (2010). 3D printing of bone substitute implants using calcium phosphate and bioactive glasses. J Eur Ceram Soc.

[CR74] Bergsma EJ, Rozema FR, Bos RRM, Debruijn WC (1993). Foreign-body reactions to resorbable poly(l-lactide) bone plates and screws used for the fixation of unstable zygomatic fractures. J Oral Maxillofac Surg.

[CR75] Bernardo JR (2010) Indirect tissue scaffold fabrication via additive manufacturing and biomimetic mineralization. Master of Science, Mechanical Engineering, The Virginia Polytechnic Institute and State University, Blacksburg, Virginia

[CR76] Bettahalli NMS, Arkesteijn ITM, Wessling M, Poot AA, Stamatialis D (2013). Corrugated round fibers to improve cell adhesion and proliferation in tissue engineering scaffolds. Acta Biomater.

[CR77] Bettinger CJ (2011). Biodegradable elastomers for tissue engineering and cell–biomaterial interactions. Macromol Biosci.

[CR78] Billiet T, Vandenhaute M, Schelfhout J, Vlierberghe SV, Dubruel P (2012). A review of trends and limitations in hydrogel-rapid prototyping for tissue engineering. Biomaterials.

[CR79] Blaker JJ, Gough JE, Maquet V, Notingher I, Boccaaccini AR (2003). In vitro evaluation of novel bioactive composites based on Bioglass^®^-filled polylactide foams for bone tissue engineering scaffolds. J Biomed Mater Res Part A.

[CR80] Blaker JJ, Maquet V, Jerome R, Boccaaccini AR, Nazhat SN (2005). Mechanical properties of highly porous PDLLA/Bioglass^®^ composite foams as scaffolds for bone tissue engineering. Acta Biomater.

[CR81] Boccaaccini AR, Notingher I, Maquet V, Jerome R (2003). Bioresorbable and bioactive composite materials based on polylactide foams filled with and coated by Bioglass^®^ particles for tissue engineering applications. J Mater Sci Mater Med.

[CR82] Boccaccini AR, Blaker JJ (2005). Bioactive composite materials for tissue engineering scaffolds. Expert Rev Med Dev.

[CR83] Bose S, Suguira S, Bandyopadhyay A (1999). Processing of controlled porosity ceramic structures via fused deposition. Scripta Mater.

[CR84] Bostman OM, Pihlajamaki HK (2000). Adverse tissue reactions to bioabsorbable fixation devices. Clin Orthop Relat Res.

[CR85] Bretcanu O, Boccaccini AR (2012). Poly-_DL_-lactic acid coated Bioglass^®^ scaffolds: toughening effects and osteosarcoma cell proliferation. J Mater Sci.

[CR86] Bretcanu O, Chen QZ, Misara SK, Boccaaccini AR, Roy I, Verne E, Brovarone CV (2007). Biodegradable polymer coated 45S5 Bioglass-derived glass-ceramic scaffolds for bone tissue engineering. Glass Technol Eur J Glass Sci Technol Part A.

[CR87] Bretcanu O, Misra SK, Roy I, Renghini C, Fiori F, Boccaccini AR (2009). In vitro biocompatibility of 45S5 Bioglass^®^-derived glass-ceramic scaffolds coated with poly(3-hydroxybutyrate). J Tissue Eng Regener Med.

[CR88] Brovarone CV, Verne E, Appendino P (2006). Macroporous bioactive glass-ceramic scaffolds for tissue engineering. J Mater Sci Mater Med.

[CR89] Brovarone CV, Verne E, Robiglio L, Martinasso G, Canuto RA, Muzio G (2008). Biocompatible glass–ceramic materials for bone substitution. J Mater Sci Mater Med.

[CR90] Brown RF, Day DE, Day TE, Jung S, Rahaman MN, Fu Q (2008). Growth and differentiation of osteoblastic cells on 13–93 bioactive glass fibers and scaffolds. Acta Biomater.

[CR91] Bruder SP, Caplan AI (2000) Bone regeneration through cellular engineering. In: Lanza RP, Lannger R, Vacanti JP (eds) Principles of tissue engineering, 2 edn. Academic Press, California, pp 683–696

[CR92] Burg KJL, Porter S, Kellam JF (2000). Biomaterial developments for bone tissue engineering. Biomaterials.

[CR93] Cao H, Kuboyama N (2010). A biodegradable porous composite scaffold of PGA/β-TCP for bone tissue engineering. Bone.

[CR94] Chai YC, Carlier A, Bolander J, Roberts SJ, Geris L, Schrooten J, Van Oosterwyck H, Luyten FP (2012). Current views on calcium phosphate osteogenicity and the translation into effective bone regeneration strategies. Acta Biomater.

[CR95] Chen QZ (2011). Foaming technology of tissue engineering scaffolds—a review. Bubble Sci Eng Technol.

[CR96] Chen QZ, Boccaccini AR (2006). Poly(d, l-lactic acid) coated 45S5 Bioglass^®^-based scaffolds: processing and characterization. J Biomed Mater Res Part A.

[CR97] Chen QZ, Thouas GA (2011). Fabrication and characterization of sol–gel derived 45S5 Bioglass^®^–ceramic scaffolds. Acta Biomater.

[CR98] Chen GQ, Wu Q (2005). The application of polyhydroxyalkanoates as tissue engineering materials. Biomaterials.

[CR99] Chen QZ, Thompson ID, Boccaaccini AR (2006). 45S5 Bioglass^®^-derived glass-ceramic scaffolds for bone tissue engineering. Biomaterials.

[CR100] Chen QZ, Efthymiou A, Salih V, Boccaccini AR (2008). Bioglass^®^-derived glass–ceramic scaffolds: study of cell proliferation and scaffold degradation in vitro. J Biomed Mater Res.

[CR101] Chen QZ, Li Y, Jin LY, Quinn JMW, Komesaroff PA (2010). A new sol–gel process for producing Na_2_O-containing bioactive glass ceramics. Acta Biomater.

[CR102] Chen QZ, Zhu CH, Thouas GA (2012). Progress and challenges in biomaterials for tissue engineering. Progr Biomater.

[CR103] Chen QZ, Zhu CH, Thouas GA (2012). Progress and challenges in biomaterials used for bone tissue engineering: bioactive glasses and elastomeric composites. Progr Biomater.

[CR104] Chen QZ, Xu JL, Yu LG, Fang XY, Khor KA (2012). Spark plasma sintering of sol–gel derived 45S5 Bioglass^®^-ceramics: mechanical properties and biocompatibility evaluation. Mater Sci Eng C.

[CR105] Choi J-W, Wicker R, Lee S-H, Choi K-H, Ha C-S, Chung I (2009). Fabrication of 3D biocompatible/biodegradable micro-scaffolds using dynamic mask projection microstereolithography. J Mater Process Technol.

[CR106] Chu T-MG, Orton DG, Hollister SJ, Feinberg SE, Halloran JW (2002). Mechanical and in vivo performance of hydroxyapatite implants with controlled architectures. Biomaterials.

[CR107] Chua CK, Leong KF, Sudarmadji N, Liu MJJ, Chou SM (2011) Selective laser sintering of functionally graded tissue scaffolds, Matrials Research Society, vol 36, pp 1006–1014

[CR108] Cooke MN, Fisher JP, Dean D, Rimnac C, Mikos AG (2002). Use of stereolithography to manufacture critical-sized 3D biodegradable scaffolds for bone ingrowth. J Biomed Mater Res B Appl Biomater.

[CR109] Crouch AS, Miller D, Luebke KJ, Hu W (2009). Correlation of anisotropic cell behaviors with topographic aspect ratio. Biomaterials.

[CR110] Cruz F, Simoes J, Coole T (2005) Direct manufacture of hydroxyapatite based bone implants by selective laser sintering. In: 2nd international conference on advanced research in virtual rapid protrotyping, Leiria, Portugal, p 119

[CR111] Daoud JT, Petropavlovskaia MS, Patapas JM, Degrandpre CE, DiRaddo RW, Rosenberg L, Tabrizian M (2011). Long-term in vitro human pancreatic islet culture using three-dimensional microfabricated scaffolds. Biomaterials.

[CR112] Dawson JI, Wahl DA, Lanham SA, Kanczler JM, Czernuszk JT, Oreffo ROC (2008). Development of specific collagen scaffolds to support the osteogenic and chondrogenic differentiation of human bone marrow stromal cells. Biomaterials.

[CR113] Day RM, Boccaccini AR, Shurey S, Roether JA, Forbes A, Hench LL, Gabe SM (2004). Assessment of polyglycolic acid mesh and bioactive glass for soft-tissue engineering scaffolds. Biomaterials.

[CR114] Dellinger JG, Cesarano J, Jamison RD (2006). Robotic deposition of model hydroxyapatite scaffolds with multi architectures and multiscale porosity for bone tissue engineering. J Biomed Mater Res.

[CR115] Devin JE, Attawin MA, Laurencin CT (1996). Three-dimensional degradable porous polymer-ceramic matrices for use in bone repair. J Biomater Sci Polym Ed.

[CR116] Dhandayuthapani B, Yoshida Y, Maekawa T, Kumar DS (2011). Polymeric scaffolds in tissue engineering application: a review. Int J Polym Sci.

[CR117] Doi Y, Kitamura S, Abe H (1995). Microbial synthesis and characterization of poly(3-hydroxyburyrate-*co*-3-hydroxyhexanoate). Macromolecules.

[CR118] Doyle C, Tanner ET, Bonfield W (1991). In vitro and in vivo evaluation of polyhydroxyburyrate and polyhydroxybutyrate reinforced with hydroxyapatite. Biomaterials.

[CR119] Duan B, Wang M (2010). Encapsulation and release of biomolecules from CaP/PHBV nanocomposite microspheres and three-dimensional scaffolds fabricated by selective laser sintering. Polym Degrad Stab.

[CR120] Elomaa L, Teixeira S, Hakala R, Korhonen H, Grijpma DW, Seppala JV (2011). Preparation of poly(ε-caprolactone)-based tissue engineering scaffolds by stereolithography. Acta Biomater.

[CR121] Elomaa L, Kokkari A, Narhi T, Seppala JV (2013). Porous 3D modeled scaffolds of bioactive glass and photocrosslinkable poly(ε-caprolactone) by stereolithography. Compos Sci Technol.

[CR122] Eosoly S, Brabazon D, Lohfeld S, Looney L (2010). Selective laser sintering of hydroxyapatite/poly-ε-caprolactone scaffolds. Acta Biomater.

[CR123] Eosoly S, Vrana NE, Lohfeld S, Hindie M, Looney L (2012). Interaction of cell culture with composite effects on the mechanical properties of polycaprolactone-hydroxypatite scaffolds fabricated via selective laser sintering (SLS). Mater Sci Eng C.

[CR124] Eshraghi S, Das S (2010). Mechanical and microstructural properties of polycaprolactone scaffolds with one-dimensional, two-dimensional, and three-dimensional orthogonally oriented porous architectures produced by selective laser sintering. Acta Biomater.

[CR125] Eslaminejad MB, Mirzadeh H, Mohamadi Y, Nickmahzar A (2007). Bone differentiation of marrow-derived mesenchymal stem cells using β-tricalcium phosphate–alginate–gelatin hybrid scaffolds. J Tissue Eng Regener Med.

[CR126] Felzmann R, Gruber S, Mitteramskogler G, Tesavibul P, Boccaccini AR, Liska R, Stampfl J (2012). Lithography-based additive manufacturing of cullular ceramic structures. Adv Eng Mater.

[CR127] Ferreira AM, Gentile P, Chiono V, Ciardelli G (2012). Collagen for bone tissue regeneration. Acta Biomater.

[CR128] Fielding GA, Bandyopadhyay A, Bose S (2012). Effects of silica and zinc oxide doping on mechanical and biological properties of 3D printed tricalcium phosphate tissue engineering scaffolds. Dent Mater.

[CR129] Fierz FC, Beckmann F, Huser M, Irsen SH, Leukers B, Witte F, Degistirici O, Andronache A, Thie M, Muller B (2008). The morphology of anisotropic 3D-printed hydroxyapatite scaffolds. Biomaterials.

[CR130] Fisher JP, Dean D, Engel PS, Mikos AG (2001). Photoinitiated polymerization of biomaterials. Annu Rev Mater Sci.

[CR131] Fu Q, Rahaman MN, Bal BS, Brown RF, Day DE (2008). Mechanical and in vitro performance of 13–93 bioactive glass scaffolds prepared by a polymer foam replication technique. Acta Biomater.

[CR132] Fu Q, Saiz E, Rahaman MN, Tomsia AP (2011). Bioactive glass scaffolds for bone tissue engineering: state of the art and future perspectives. Mater Sci Eng C.

[CR133] Fukasawa T, Deng ZY, Ando M, Ohji T, Goto Y (2001). Pore structure of porous ceramics synthesized from water-based slurry by freeze-dry process. J Mater Sci.

[CR134] Ge Z, Wang L, Heng CB, Tian X-F, Lu K, Fan VTW, Yeo JF, Cao T, Tan E (2009). Proliferation and differentiation of human osteoblasts within 3D printed poly-lactic-*co*-glycolic acid scaffolds. J Biomater Appl.

[CR135] Gerhardt L-C, Boccaccini AR (2010). Bioactive glass and glass-ceramic scaffolds for bone tissue engineering. Materials.

[CR136] Gollwitzer H, Ibrahim K, Meyer H, Mittelmeier W, Busch R, Stemberger A (2003). Antibacterial poly(d, l-lactic acid) coating of medical implants using a biodegradable drug delivery technology. J Antimicrob Chemother.

[CR137] Gollwitzer H, Thomas P, Diehl P, Steinhauser E, Summer B, Barnstorf S (2005). Biomechanical and allergological characteristics of a biodegradable poly(d, l-lactic acid) coating for orthopaedic implants. J Orthoped Res.

[CR138] Goodridge RD (2004) Indirect selective laser sintering of an apatite-mullite glass–ceramic. PhD School of Mechanical Engineering, University of Leeds 10.1243/095441105X69051 16459446

[CR139] Goodridge RD, Wood DJ, Ohtsuki C, Dalgarno KW (2007). Biological evaluation of an apatite–mullite glass-ceramic produced via selective laser sintering. Acta Biomater.

[CR140] Guan L, Davies JE (2004). Preparation and characterization of a highly macroporous biodegradable composite tissue engineering scaffold. J Biomed Mater Res Part A.

[CR141] Harris LD, Kim B-S, Mooney DJ (1998). Open pore biodegradable matrices formed with gas foaming. J Biomed Mater Res.

[CR142] Hattiangadi A, Bandyopadhyay A (2000). Modeling of multiple pore ceramic materials fabricated via fused deposition process. Scripta Mater.

[CR143] Haugen H, Will J, Kohler A, Hopfner U, Aigner J, Wintermantel E (2004). Ceramic TiO_2_-foams: characterisation of a potential scaffold. J Eur Ceram Soc.

[CR144] Hayati AN, Rezaie HR, Hosseinalipour SM (2011). Preparation of poly(3-hydroxybutyrate)/nano-hydroxyapatite composite scaffolds for bone tissue engineering. Mater Lett.

[CR145] Heller C, Schwentenwein M, Russmueller G, Varga F, Stampfl J, Liska R (2009). Vinyl esters: low cytotoxicity monomers for the fabrication of biocompatible 3D scaffolds by lithography based additive manufacturing. J Polym Sci Part A Polym Chem.

[CR146] Hench LL (1998). Bioceramics. J Am Ceram Soc.

[CR147] Hench LL (2006). The story of Bioglass^®^. J Mater Sci Mater Med.

[CR148] Hench LL, Wilson J (1999). An Introduction to bioceramics.

[CR149] Hench LL, Splinter RJ, Allen WC (1971). Bonding mechanisms at the interface of ceramic prosthetic materials. J Biomed Mater Res Symp.

[CR150] Heo S-J, Kim S-E, Wei J, Hyun Y-T, Yun H-S, Kim D-H, Shin JW, Shin J-W (2009). Fabrication and characterization of novel nano-and micro-HA/PCL composite scaffolds using a modified rapid prototyping process. J Biomed Mater Res.

[CR151] Hoelzle DJ, Alleyne AG, Johnson AJW (2008). Micro-robotic deposition guidelines by a design of experiments approach to maximize fabrication reliability for the bone scaffold application. Acta Biomater.

[CR152] Hoque ME, Chuan YL, Pashby I (2011). Extrusion based rapid prototyping technique: an advanced platform for tissue engineering scaffold fabrication. Biopolymers.

[CR153] Hsu S-H, Yen H-J, Tseng C-S, Cheng C-S, Tsai C-L (2007). Evaluation of the growth of chondrocytes and osteoblasts seeded into precision scaffolds fabricated by fused deposition manufacturing. J Biomed Mater Res B Appl Biomater.

[CR154] Hutmacher DW (2000). Scaffolds in tissue engineering bone and cartilage. Biomaterials.

[CR155] Hutmacher DW, Cool S (2007). Concepts of scaffold-based tissue engineering-the rationale to use solid free-form fabrication techniques. J Cell Mol Med.

[CR156] Hutmacher DW, Schantz T, Zein I, Ng KW, Teoh SH, Tan KC (2001). Mechanical properties and cell cultural response of polycaprolactone scaffolds designed and fabricated via fused deposition modeling. J Biomed Mater Res.

[CR157] Hutmacher DW, Sittinger M, Risbud MV (2004). Scaffold-based tissue engineering: rationale for computer-aided design and solid free-form fabrication systems. Trends Biotechnol.

[CR158] Ishizaki K, Komarneni S, Nanko M (1998). Porous materials: processing technology and applications.

[CR159] Iyer S, McIntosh J, Bandyopadhyay A, Langrana N, Safari A, Danforth SC, Clancy RB, Gasdaska C, Whalen PJ (2008). Microstructural characterization and mechanical properties of Si_3_N_4_ fomed by fused deposition of ceramics. Int J Appl Ceram Technol.

[CR160] Jones JR (2013). Review of bioactive glass: from Hench to hybrids. Acta Biomater.

[CR161] Kalita SJ, Bose S, Hosick HL, Bandyopadhyay A (2003). Development of controlled porosity polymer–ceramic composite scaffolds via fused deposition modeling. Mater Sci Eng C.

[CR162] Kanczler JM, Mirmalek-Sani S-H, Hanley NA, Ivanov AL, Barry JJA, Upton C, Shakesheff KM, Howdle SM, Antonov EN, Bagratashvili VN, Popov VK, Oreffo ROC (2009). Biocompatibility and osteogenic potential of human fetal femur-derived cells on surface selective laser sintered scaffolds. Acta Biomater.

[CR163] Kemppainen JM, Hollister SJ (2010). Tailoring the mechanical properties of 3D-designed poly(glycerol sebacate) scaffolds for cartilage applications. J Biomed Mater Res Part A.

[CR164] Khoda AKMB, Ozbolat IT, Koc B (2010) Engineered tissue scaffolds with variational porous architecture. J Biomech Eng 133:011001 10.1115/1.4002933 21186891

[CR165] Kim JY, Cho D-W (2009). The optimization of hybrid scaffold fabrication process in precision deposition system using design of experiments. Microsyst Technol.

[CR166] Kim JY, Cho D-W (2009). Blended PCL/PLGA scaffold fabrication using multi-head deposition system. Microelectron Eng.

[CR167] Kim SS, Utsunomiya H, Koski JA, Wu BM, Cima MJ, Sohn J, Mukai K, Grifith L, Vacanti JP (1998). Survival and function of hepatocytes on a novel three-dimensional synthetic biodegradable polymer scaffold with an intrinsic network of channels. Ann Surg.

[CR168] Kim HW, Lee SY, Bae CJ, Noh YJ, Kim HE, Kim HM, Ko JS (2003). Porous ZrO_2_ bone scaffold coated with hydroxyapatite with fluorapatite intermediate layer. Biomaterials.

[CR169] Kim HD, Bae EH, Kwon IC, Pal RR, Nam JD, Lee DS (2004). Effect of PEG–PLLA diblock copolymer on macroporous PLLA scaffoldsbythermallyinducedphaseseparation. Biomaterials.

[CR170] Kim JY, Jin G-Z, Park IS, Kim J-N, Chun SY, Park EK, Kim S-Y, Yoo J, Kim S-H, Rhie J-W, Cho D-W (2010). Evaluation of solid free-form fabrication-based scaffolds seeded with osteoblasts and human umbilical vein endothelial cells for use in vivo osteogenesis. Tissue Eng Part A.

[CR171] Kolan KCR, Leu MC, Hilmas GE, Velez M (2012). Effect of material, process parameters, and simulated body fluids on mechanical properties of 13-93 bioactive glass porous constructs made by selective laser sintering. J Mech Behav Biomed Mater.

[CR172] Korpela J, Kokkari A, Korhonen H, Malin M, Narhi T, Seppala J (2013). Biodegradable and bioactive porous scaffold structures prepared using fused deposition modeling. J Biomed Mater Res B Appl Biomater.

[CR173] Kruth JP, Wang X, Laoui T, Froyen L (2003). Lasers and materials in selective laser sintering. Assembly Autom.

[CR174] Lam CXF, Hutmacher DW, Schantz J-T, Woodruff MA, Teoh SH (2009). Evaluation of polycaprolactone scaffold degradation for 6 months in vitro and in vivo. J Biomed Mater Res.

[CR175] Landers R, Pfister A, Hubner U, John H, Schmelzeisen R, Mulhaupt R (2002). Fabrication of soft tissue engineering scaffolds by means of rapid prototyping techniques. J Mater Sci.

[CR176] Landers R, Hubner U, Schmelzeisen R, Mulhaupt R (2002). Rapid prototyping of scaffolds derived from thermoreversible hydrogels and tailored for applications in tissue engineering. Biomaterials.

[CR177] Langer R, Vacanti JP (1993). Tissue engineering. Science.

[CR178] Langer R, Vacanti JP, Vacanti CA, Atala A, Freed LE, Vunjak-Novakovic G (1995). Tissue engineering biomedical applications. Tissue Eng.

[CR179] Laurencin CT, Attawin MA, Elgendy HE, Herbert KM (1996). Tissue engineered bone-regeneration using degradable polymers: the formation of mineralized matrices. Bone.

[CR180] Lee KW, Wang SF, Fox BC, Ritman EL, Yaszemski MJ, Lu LC (2007). Poly(propylene fumalate) bone tissue engineering scaffold fabrication using stereolithography: effects of resin formulations and laser parameters. Biomacromolecules.

[CR181] Lee JW, Lan PX, Kim B, Lim G, Dong-Woo C (2008). Fabrication and characteristic analysis of a poly(propylene fumate) scaffold using micro-stereolithography technology. J Biomed Mater Res B Appl Biomater.

[CR182] Lee K-S, Kim RH, Yang D-Y, Park SH (2008). Advances in 3D nano/microfabrication using two-photon initiated polymerization. Prog Polym Sci.

[CR183] Lee J-S, Cha HD, Shim J-H, Jung JW, Kim JY, Cho D-W (2012). Effect of pore architecture and stacking direction on mechanical properties of solid freeform fabrication-based scaffold for bone tissue engineering. J Biomed Mater Res Part A.

[CR184] Leong KF, Cheah CM, Chua CK (2003). Solid freeform fabrication of three-dimensional scaffolds for engineering replacement tissues and organs. Biomaterials.

[CR185] Leong DT, Gupta A, Bai HF, Wan G, Yoong LF, Too H-P, Chew FT, Hutmacher DW (2007). Absolute quantification of gene expression in biomaterials research using real-time PCR. Biomaterials.

[CR186] Li HY, Chang J (2004). Preparation and characterization of bioactive and biodegradable Wollastonite/poly(d, l-lactic acid) composite scaffolds. J Mater Sci Mater Med.

[CR187] Li HY, Du RL, Chang J (2005). Fabrication, characterization, and in vitro degradation of composite scaffolds based on PHBV and bioactive glass. J Biomater Appl.

[CR188] Li J, Zhang L, Lv S, Li S, Wang N, Zhang Z (2011). Fabrication of individual scaffolds based on a patient-specific alveolar bone defect model. J Biotechnol.

[CR189] Li Y, Cook WD, Moorhoff C, Huang WC, Chen QZ (2013). Synthesis, characterization and properties of biocompatible poly(glycerol sebacate) pre-polymer and gel. Polym Int.

[CR190] Li Z, Chen X, Zhao N, Dong H, Li Y, Lin C (2013). Stiff macro-porous bioactive glasse ceramic scaffold: fabrication by rapid prototyping template, characterization and in vitro bioactivity. Mater Chem Phys.

[CR191] Liu X, Huang W, Fu H, Yao A, Wang D, Pan H, Lu WW (2009). Bioactive borosilicate glass scaffolds: improvement on the strength of glass-based scaffolds for tissue engineering. J Mater Sci Mater Med.

[CR192] Liu L, Xiong Z, Yan Y, Zhang R, Wang X, Jin L (2009). Multinozzle low-temperature deposition system for construction of gradient tissue engineering scaffolds. J Biomed Mater Res B Appl Biomater.

[CR193] Liu Y, Lim JL, Teoh S-H (2013). Review: development of clinically relevant scaffolds for vascularised bone tissue engineering. Biotechnol Adv.

[CR194] Lohfeld S, Cahill S, Barron V, McHugh P, Dürselen L, Kreja L, Bausewein C, Ignatius A (2012). Fabrication, mechanical and in vivo performance of polycaprolactone/tricalcium phosphate composite scaffolds. Acta Biomater.

[CR195] Lorrison J, Dalgarno K, Wood D (2005). Processing of an apatite-mullite glass-ceramic and an hydroxyapatite/phosphate glass composite by selective laser sintering. J Mater Sci Mater Med.

[CR196] Lu HH, El-Amin SF, Scott KD, Laurencin CT (2003). Three-dimensional, bioactive, biodegradable, polymer-bioactive glass composite scaffolds with improved mechanical properties support collagen synthesis and mineralization of human osteoblast-like cells in vitro. J Biomed Mater Res Part A.

[CR197] Mano JF, Sousa RA, Boesel LF, Neves NM, Reis RL (2004). Bioinert, biodegradable and injectable polymeric matrix composites for hard tissue replacement: state of the art and recent developments. Compos Sci Technol.

[CR198] Maquet V, Boccaaccini AR, Pravata L, Notingher I, Jerome R (2003). Preparation, characterization, and in vitro degradation of bioresorbable and bioactive composites based on Bioglass^®^-filled polylactide foams. J Biomed Mater Res Part A.

[CR199] Maquet V, Boccaccinic AR, Pravata L, Notingher I, Jerome R (2004). Porous poly(alpha-hydroxyacid)/Bioglass^®^ composite scaffolds for bone tissue engineering. I: preparation and in vitro characterisation. Biomaterials.

[CR200] Marcacci M, Kon E, Moukhachev V, Lavroukov A, Kutepov S, Quarto R (2007). Stem cells associated with macroporous bioceramics for long bone repair: 6- to 7-year outcome of a pilot clinical study. Tissue Eng.

[CR201] Martin C, Winet H, Bao JY (1996). Acidity near eroding polylactide–polyglycolide in vitro and in vivo rabbit tibial bone chambers. Biomaterials.

[CR202] Martínez-Vázquez FJ, Perera FH, Miranda P, Pajares A, Guiberteau F (2010). Improving the compressive strength of bioceramic robocast scaffolds by polymer infiltration. Acta Biomater.

[CR203] Melchels FPW, Feijen J, Grijpma DW (2009). A poly(d, l-lactide) resin for the preparation of tissue engineering scaffolds by stereolithography. Biomaterials.

[CR204] Melchels FPW, Feijen J, Grijpma DW (2010). A review on stereolithography and its applications in biomedical engineering. Biomaterials.

[CR205] Melchels FPW, Domingos MAN, Klein TJ, Malda J, Bartolo PJ, Hutmacher DW (2012). Additive manufacturing of tissues and organs. Prog Polym Sci.

[CR206] Meszaros R, Zhao R, Travitzky NA, Fey T, Greil P, Wondraczek L (2011). Three-dimensional printing of a bioactive glass. Glass Technol.

[CR207] Metze A-L, Grimm A, Nooeaid P, Roether JA, Hum J, Newby PJ, Schubert DW, Boccaccini AR (2013). Gelatin coated 45S5 Bioglass^®^-derived scaffolds for bone tissue engineering. Key Eng Mater.

[CR208] Middleton JC, Tipton AJ (2000). Synthetic biodegradable polymers as orthopedic devices. Biomaterials.

[CR209] Mikos AG, Temenoff JS (2000). Formation of highly porous biodegradable scaffolds for tissue engineering. Electron J Biotechnol.

[CR210] Miranda P, Saiz E, Gryn K, Tomsia AP (2006). Sintering and robocasting of β-tricalcium phosphate scaffolds for orthopaedic applications. Acta Biomater.

[CR211] Miranda P, Pajares A, Guiberteau F (2008). Finite element modeling as a tool for predicting the fracture behavior of robocast scaffolds. Acta Biomater.

[CR212] Misra SK, Valappil SP, Roy I, Boccaaccini AR (2006). Polyhydroxyalkanoate (PHA)/inorganic phase composites for tissue engineering applications. Biomacromolecules.

[CR213] Molladavoodi S, Gorbet M, Medley J, Kwon HJ (2013). Investigation of microstructure, mechanical properties and cellular viability of poly(_L_-lactic acid) tissue engineering scaffolds prepared by different thermally induced phase separation protocols. J Mech Behav Biomed Mater.

[CR214] Montanaro L, Jorand Y, Fantozzi G, Negro A (1998). Ceramic foams by powder processing. J Eur Ceram Soc.

[CR215] Muzzarelli RAA, Zucchini C, Ilari P, Pugnaloni A, Belmonte MM, Biagini G, Castaldini C (1993). Osteoconductive properties of methlpyrrolidinone chitosan in an animal-model. Biomaterials.

[CR216] Nam YS, Park TG (1999). Biodegradable polymeric microcellular foams by modified thermally induced phase separation method. Biomaterials.

[CR217] Navarro M, Ginebra MP, Planell JA (2004). Development and cell response of a new biodegradable composite scaffold for guided bone regeneration. J Mater Sci Mater Med.

[CR218] Oliveira AL, Sousa EC, Silva NA, Sousa N, Salgada AJ, Reis RL (2010). Peripheral mineralization of a 3D biodegradable tubular construct as a way to enhance guidance stabilization in spinal cord injury regeneration. J Mater Sci Mater Med.

[CR219] Pham QP, Sharma U, Mikos AG (2006). Electrospinning of polymeric nanofibers for tissue engineering applications: a review. Tissue Eng.

[CR220] Pham DT, Dotchev KD, Yusoff WAY (2008). Deterioration of polyamide powder properties in the laser sintering process. Proceedings of The Institution of Mechanical Engineers, Part C. J Mech Eng Sci.

[CR221] Pitt CG, Gratzel MM, Kimmel GL (1981). Aliphatic polyesters. 2. The degradation of poly(_DL_-lactide), poly(ε-caprolactone) and their copolymers in vivo. Biomaterials.

[CR222] Potijanyakul P, Sattayasansakul W, Pongpanich S, Leepong N, Kintarak S (2010). Effects of enamel matrix derivative on bioactive glass in rat calvarium defects. J Oral Implantol.

[CR223] Puppi D, Chiellini F, Piras AM, Chiellini E (2010). Polymeric materials for bone and cartilage repair. Prog Polym Sci.

[CR224] Ramanath HS, Chua CK, Leong KF, Shah KD (2008). Melt flow behaviour of poly-ε-caprolactone in fused deposition modeling. J Mater Sci Mater Med.

[CR225] Ramay HR, Zhang MQ (2003). Preparation of porous hydroxyapatite scaffolds by combination of the gel-casting and polymer sponge methods. Biomaterials.

[CR226] Rattanakit P, Moulton SE, Santiago KS, Liawruangrath S, Wallace GG (2012). Extrusion printed polymer structures: a facile and versatile approach to tailored drug delivery platforms. Int J Pharm.

[CR227] Raucci MG, Guarino V, Ambrosio L (2010). Hybrid composite scaffolds prepared by sol–gel method for bone regeneration. Compos Sci Technol.

[CR228] Reed JS (1988). Principles of ceramic synthesis.

[CR229] Rezwan K, Chen QZ, Blaker JJ, Boccaccini AR (2006). Biodegradable and bioactive porous polymer/inorganic composite scaffolds for bone tissue engineering. Biomaterials.

[CR230] Rich J, Jaakkola T, Tirri T, Narhi T, Yli-Urpo A, Seppala J (2002). In vitro evaluation of poly(ε-caprolactone-co-d,l-lactide)/bioactive glass composites. Biomaterials.

[CR231] Roether JA, Gough JE, Boccaccini AR, Hench LL, Maquet V, Jerome R (2002). Novel bioresorbable and bioactive composites based on bioactive glass and polylactide foams for bone tissue engineering. J Mater Sci Mater Med.

[CR232] Roy TD, Simon JL, Ricci JL, Rekow ED, Thompson VP, Parsons JR (2003). Performance of hydroxyapatite bone repair scaffolds created via three-dimensional fabrication techniques. J Biomed Mater Res Part A.

[CR233] Russell JL, Block JE (1999). Clinical utility of demineralized bone matrix for osseous defects, arthrodesis, and reconstruction: impact of processing techniques and study methodology. Orthopedics.

[CR234] Russias J, Saiz E, Deville S, Gryn K, Liu G, Nalla RK, Tomsia AP (2007). Fabrication and in vitro characterization of three-dimensional organic/inorganic scaffolds by robocasting. J Biomed Mater Res.

[CR235] Santos CFL, Siilva AP, Lopes L, Pires I, Correia IJ (2012). Design and production of sintered β-tricalcium phosphate 3D scaffolds for bone tissue regeneration. Mater Sci Eng C.

[CR236] Schmidmaier G, Wildemann B, Bail H, Lucke M, Fuchs T, Stemberger A (2001). Local application of growth factors (insulin-like growth factor-1 and transforming growth factor-beta 1) from a biodegradable poly(d,l-lactide) coating of osteosynthetic implants accelerates fracture healing in rats. Bone.

[CR237] Schmidmaier G, Wildemann B, Stemberger A, Haas NP, Raschke M (2001). Biodegradable poly(d, l-lactide) coating of implants for continuous release of growth factors. J Biomed Mater Res.

[CR238] Seck TM, Melchels FPW, Feijen J, Grijpma DW (2010). Designed biodegradable hydrogel structures prepared by stereolithography using poly(ethylene glycol)/poly(d, l-lactide)-based resins. J Controlled Release.

[CR239] Seitz H, Rieder W, Irsen S, Leukers B, Tille C (2005). Three-dimensional printing of porous ceramic scaffolds for bone tissue engineering. J Biomed Mater Res B Appl Biomater.

[CR240] Seol YJ, Park DY, Park JY, Kim SW, Park SJ, Cho DW (2013). A new method of fabricating robust freeform 3D ceramic scaffolds for bone tissue regeneration. Biotechnol Bioeng.

[CR241] Seppala J, Korhonen H, Hakala R, Malin M (2011). Photocrosslinkable polyesters and poly(ester anhydride)s for biomedical applications. Macromol Biosci.

[CR242] Sepulveda P, Jones JR, Hench LL (2002). Bioactive sol–gel foams for tissue repair. J Biomed Mater Res.

[CR243] Serra T, Planell JA, Navarro M (2013). High-resolution PLA-based composite scaffolds via 3-D printing technology. Acta Biomater.

[CR244] Shanjani Y, Hu Y, Pilliar RM, Toyserkani E (2011). Mechanical characteristics of solid-freeform-fabricated porous calcium polyphosphate structures with oriented stacked layers. Acta Biomater.

[CR245] Sharaf B, Faris CB, Abukawa H, Susarla SM, Vacanti JP, Kaban LB, Troulis MJ (2012). Three-dimensionally printed polycaprolactone and β-tricalcium phosphate scaffolds for bone tissue engineering: an in vitro study. J Oral Maxillofac Surg.

[CR246] Sharifi S, Kamali M, Mohtaram NK, Shokrgozar MA, Rabiee SM, Atai M, Imani M, Mirzadeh H (2011). Preparation, mechanical properties, and in vitro biocompatibility of novel nanocomposites based on polyhexamethylene carbonate fumarate and nanohydroxyapatite. Polym Adv Technol.

[CR247] Sherwood JK, Riley SL, Palazzolo R, Brown SC, Monkhouse DC, Coates M, Griffith LG, Landeen LK, Ratcliffe A (2002). A three-dimensional osteochondral composite scaffold for articular cartilage repair. Biomaterials.

[CR248] Shokrollahi P, Mirzadeh H, Scherman OA, Huck WTS (2010). Biological and mechanical properties of novel composites based on supramolecular polycaprolactone and functionalised hydroxyapatite. J Biomed Mater Res A.

[CR249] Shor L, Guceri S, Wen X, Gandhi M, Sun W (2007). Fabrication of three-dimensional polycaprolactone/hydroxyapatite tissue scaffolds and osteoblast-scaffold interactions in vitro. Biomaterials.

[CR250] Shor L, Guceri S, Chang R, Gordon J, Kang Q, Hartsock L, An Y, Sun W (2009). Precision extruding deposition (PED) fabrication of polycaprolactone (PCL) scaffolds for bone tissue engineering. Biofabrication.

[CR251] Shuai C, Zhuang J, Hu H, Peng S, Liu D, Liu J (2013). *In vitro* bioactivity and degradability of β-tricalcium phosphate porous scaffold fabricated via selective laser sintering. Biotechnol Appl Biochem.

[CR252] Simon JL, Roy TD, Parsons JR, Rekow ED, Thompson VP, Kemnitzer J, Ricci JL (2003). Engineered cellular response to scaffold architecture in a rabbit trephine defect. J Biomed Mater Res Part A.

[CR253] Smay JE, Cesarano J, Lewis JA (2002). Colloidal inks for directed assembly of 3-D periodic structures. Langmuir.

[CR254] Sobral JM, Caridade SG, Sousa RA, Mano JF, Reis RL (2011). Three-dimentional plotted scaffolds with controlled pore size gradients: effect of scaffold geometry on mechnical performance and cell seeding efficiency. Acta Biomater.

[CR255] Stamboulis AG, Boccaaccini AR, Hench LL (2002). Novel biodegradable polymer/bioactive glass composites for tissue engineering applications. Adv Eng Mater.

[CR256] Sudarmadji N, Tan JY, Leong KF, Chua CK, Loh YT (2011). Investigation of the mechanical properties and porosity relationships in selective laser-sintered polyhedral for functionally graded scaffolds. Acta Biomater.

[CR257] Sun J-Y, Yang Y-S, Zhong J, Greenspan DC (2007). The effect of the ionic products of Bioglass^®^ dissolution on human osteoblasts growth cycle in vitro. J Tissue Eng Regener Med.

[CR258] Suuronen R, Pohjonen T, Hietanen J, Lindquist C (1998). A 5-year in vitro and in vivo study of the biodegradation of polylactide plates. J Oral Maxillofac Surg.

[CR259] Tam J, Rozema FR, Bos RRM, Roodenburg JLN, Nikkels PGJ, Vermey A (1996). Poly(_L_-lactide) bone plates and screws for internal fixation of mandibular swing osteotomies. Int J Oral Maxillofac Surg.

[CR260] Tatakis DN, Trombelli L (1999). Adverse effects associated with a bioabsorbable guided tissue regeneration device in the treatment of human gingival recession defects: a clinicopathologic case report. J Periodontol.

[CR261] Tellis BC, Szivek JA, Bliss CL, Margolis DS, Vaidyanathan RK, Calvert P (2008). Trabecular scaffolds created using micro CT guided fused deposition modeling. Mater Sci Eng C.

[CR262] Tesavibul P, Felzmann R, Bruber S, Liska R, Thompson I, Boccaaccini AR, Stampfl J (2012). Processing of 45S5 Bioglass^®^ by lithography-based additive manufacturing. Mater Lett.

[CR263] Thein-Han WW, Misra RDK (2009). Biomimetic chitosan–nanohydroxyapatite composite scaffolds for bone tissue engineering. Acta Biomater.

[CR264] Tian H, Tang Z, Zhuang X, Chen X, Jing X (2012). Biodegradable synthetic polymers: preparation, functionalization and biomedical application. Prog Polym Sci.

[CR265] Tirella A, Vozzi F, Vozzi G, Ahluwalia A (2011). PAM2 (Piston Assisted Microsyringe): a new rapid prototyping technique for biofabrication of cell incorporated scaffolds. Tissue Eng Part C.

[CR266] Tulliani J-M, Lombardi M, Palmero P, Fornabaio M, Gibson LJ (2013). Development and mechanical characterization of novel ceramic foams fabricated by gel-casting. J Eur Ceram Soc.

[CR267] Verma S, Bhatia Y, Valappil SP, Roy I (2002). A possible role of poly-3-hydroxybutyric acid in antibiotic production in streptomyces. Arch Microbiol.

[CR268] Verrier S, Blaker JJ, Maquet V, Hench LL, Boccaaccini AR (2004). PDLLA/Bioglass^®^ composites for soft-tissue and hard-tissue engineering: an in vitro cell biology assessment. Biomaterials.

[CR269] Vozzi G, Ahluwalia A (2007). Microfabrication for tissue engineering: rethinking the cells-on-a scaffold approach. J Mater Chem.

[CR270] Vozzi G, Previti A, Rossi DD, Ahluwalia A (2002). Microsyringe-based deposition of two-dimensional and three-dimensional polymer scaffolds with a well-defined geometry for application to tissue engineering. Tissue Eng.

[CR271] Vozzi G, Flaim C, Ahluwalia A, Bthatia S (2003). Fabrication of PLGA scaffolds using soft lithography and microsyringe deposition. Biomaterials.

[CR272] Vozzi G, Corallo C, Daraio C (2013). Pressure-activated microsyringe composite scaffold of poly(l-lactic acid) and carbon nanotubes for bone tissue engineering. J Appl Polym Sci.

[CR273] Wang Y, Ameer GA, Sheppard BJ, Langer R (2002). A tough biodegradable elastomer. Nat Biotechnol.

[CR274] Wang F, Shor L, Darling A, Khalil S, Sun W, Güçeri S, Lau A (2004). Precision extruding deposition and characterization of cellular poly-ε-caprolactone tissue scaffolds. Rapid Prototyp J.

[CR275] Warnke PH, Seitz H, Warnke F, Becker ST, Sivananthan S, Sherry E, Liu Q, Wiltfang J, Douglas T (2010). Ceramic scaffolds produced by computer-assisted 3D printing and sintering: characterization and biocompatibility investigations. J Biomed Mater Res B Appl Biomater.

[CR276] Weiss T, Hildebrand G, Schade R, Liefeith K (2009). Two-photon polymerization for microfabrication of three-dimensional scaffolds for tissue engineering application. Eng Life Sci.

[CR277] Weiss T, Schade R, Laube T, Berg A, Hildebrand G, Wyrwa R, Schnabelrauch M, Liefeith K (2011). Two-photon polymerization of biocompatible photopolymers for microstructured 3D biointerfaces. Adv Eng Mater.

[CR278] Williams JM, Adewunmi A, Schek RM, Flanagan CL, Krebsbach PH, Feinberg SE, Hollister SJ, Das S (2005). Bone tissue engineering using polycaprolactone scaffolds fabricated via selective laser sintering. Biomaterials.

[CR279] Winkel A, Meszaros R, Reinsch S, Muller R, Travizky N, Fey T, Greil P, Wondraczek L (2012). Sintering of 3D-printed glass/HAp composites. J Am Ceram Soc.

[CR280] Wiria FE, Leong KF, Chua CK, Liu Y (2007). Poly-ε-caprolactone/hydroxyapatite for tissue engineering scaffold fabrication via selective laser sintering. Acta Biomater.

[CR281] Wojtowicz AM, Shekaran A, Oest ME, Dupont KM, Templeman KL, Hutmacher DW, Guldberg RE, Garcıa AJ (2010). Coating of biomaterial scaffolds with the collagen-mimetic peptide GFOGER for bone defect repair. Biomaterials.

[CR282] Woodruff MA, Hutmacher DW (2010). The return of a forgotten polymer-polycaprolactone in the 21st century. Prog Polym Sci.

[CR283] Wu ZY, Hill RG, Yue S, Nightingale D, Lee PD, Jones JR (2011). Melt-derived bioactive glass scaffolds produced by a gel-cast foaming technique. Acta Biomater.

[CR284] Xiong Z, Yan Y, Wang S, Zhang R, Zhang C (2002). Fabrication of porous scaffolds for bone tissue engineering via low-temperature deposition. Scripta Mater.

[CR285] Xynos ID, Edgar AJ, Buttery LDK, Hench LL, Polak M (2001). Gene expression profiling of human osteoblasts following treatment with the ionic products of Bioglass^®^ 45S5 dissolution. J Biomed Mater Res.

[CR286] Yan Y, Xiong Z, Hu Y, Wang S, Zhang R, Zhang C (2003). Layered manufacturing of tissue engineering scaffolds via multi-nozzle deposition. Mater Lett.

[CR287] Yen H-J, Tseng C-S, Hsu S-H, Tsai C-L (2009). Evaluation of chondrocyte growth in the highly porous scaffolds made by fused deposition manufacturing (FDM) filled with type II collagen. Biomed Microdev.

[CR288] Yeong W-Y, Chua C-K, Leong K-F, Chandrasekaran M (2004). Rapid prototyping in tissue engineering: challenges and potential. Trends Biotechnol.

[CR289] Yeong WY, Sudarmadji N, Yu HY, Chua CK, Leong KF, Venkatraman SS, Boey YCF, Tan LP (2010). Porous polycaprolactone scaffold for cardiac tissue engineering fabricated by selective laser sintering. Acta Biomater.

[CR290] Yin Y, Ye F, Cui J, Zhang F, Li X, Yao K (2003). Preparation and characterization of macroporous chitosan–gelatin/β-tricalcium phosphate composite scaffolds for bone tissue engineering. J Biomed Mater Res Part A.

[CR291] Zein I, Hutmacher DW, Tan KC, Teoh SH (2002). Fused deposition modeling of novel scaffold architectures for tissue engineering applications. Biomaterials.

[CR292] Zhang K, Wang Y, Hillmyer MA, Francis LF (2004). Processing and properties of porous poly(_L_-lactide)/bioactive glass composites. Biomaterials.

[CR293] Zhang Y, Hao L, Savalani MM, Harris RA, Silvio LD, Tanner KE (2008). *In vitro* biocompatibility of hydroxyapatite-reinforced polymeric composites manufactured by selective laser sintering. J Biomed Mater Res.

[CR294] Zhou Y, Hutmacher DW, Varawan S-L, Lim TM (2007). *In vitro* bone engineering based on polycaprolactone and polycaprolactone–tricalcium phosphate composites. Polym Int.

[CR295] Zhou Y, Chen F, Ho ST, Woodruff MA, Lim TM, Hutmacher DW (2007). Combined marrow stromal cell-sheet techniques and high-strength biodegradable composite scaffolds for engineered functional bone grafts. Biomaterials.

